# Supplementary material for: Computational enzyme design by catalytic motif scaffolding
Source: Nature. 2025 Dec 3;649(8095):237–45. doi: 10.1038/s41586-025-09747-9 (PMC12727513; doi:10.1038/s41586-025-09747-9)
Supplement: Supplementary file 1 — This file contains Supplementary Methods, Supplementary Figs. 1–23 and Supplementary Tables 1–9. Supplementary Methods detail the Riff-Diff design workflow, equations and rationale for the stability model and the active site metric, and all models used to fit kinetic data. The supplementary figures present computational metrics of designed enzymes and supporting experimental data, including Michaelis–Menten plots, CD, SAXS and HPLC experiments and NMR spectra. The supplementary tables list sequences for all ordered designs, kinetic parameters of the designs and previously reported designs, primers and mass spectrometry results. [file 41586_2025_9747_MOESM1_ESM.docx]

Supplementary Materials for

**Computational enzyme design by catalytic motif scaffolding**

Markus Braun^1^#, Adrian Tripp^1^#, Morakot Chakatok^1^, Sigrid Kaltenbrunner^1^, Celina Fischer^1^, David Stoll^1^, Aleksandar Bijelic^1^, Wael Elaily^1^, Massimo G. Totaro^1^, Melanie Moser^1^, Shlomo Y. Hoch^1,2^, Horst Lechner^1^, Federico Rossi^3^, Matteo Aleotti^3^, Mélanie Hall^3,4,5^, Gustav Oberdorfer^1,5^*

**Affiliations:**

^1^Institute of Biochemistry, Graz University of Technology; Petersgasse 12/2, 8010 Graz, Austria

^2^Weizmann Institute of Science; Herzl St 234, Rhovolt, Israel

^3^Institute of Chemistry, University of Graz; Heinrichstrasse 28, 8010 Graz, Austria

^4^BioHealth, Field of Excellence, University of Graz, Austria

^5^BioTechMed Graz, Mozartgasse 12/2, 8010 Graz, Austria

*Corresponding author. Email: gustav.oberdorfer@tugraz.at

#These authors contributed equally to this work.

**Content**

[Supplementary Methods 3](#_Toc208828285)

[The Riff-Diff pipeline 3](#_Toc208828286)

[Prediction of stability of designed retro-aldolases 7](#_Toc208828287)

[AlphaFold3 active site metric 7](#_Toc208828288)

[Design of MBH constructs 8](#_Toc208828289)

[Fitting of pKa values for RAD constructs 9](#_Toc208828290)

[Substrate inhibition 9](#_Toc208828291)

[Fitting of Michaelis-Menten kinetics for MBH constructs 9](#_Toc208828292)

[Supplementary figures 10](#_Toc208828293)

[Fig. S1. Computational metrics of selected RAD models. 10](#_Toc208828294)

[Fig. S2. Mutation of active site lysine residues to alanine leads to significant loss of activity of designed retro-aldolases. 11](#_Toc208828295)

[Fig. S3. CD spectra of designed RAD enzymes. 12](#_Toc208828296)

[Fig. S4. Dimensionless Kratky plots 13](#_Toc208828297)

[Fig. S5. Michaelis Menten kinetics of RADs 14](#_Toc208828298)

[Fig. S6. Proposed reaction mechanism for the RA98.5-8F-catalyzed retro-aldol reaction of methodol. 15](#_Toc208828299)

[Fig. S7. Michaelis-Menten kinetics of RAD variants 16](#_Toc208828300)

[Fig. S8. pH profiles of RADs. 17](#_Toc208828301)

[Fig. S9. Chemical denaturation of RAD designs 18](#_Toc208828302)

[Fig. S10. Individual correlations of computational metrics used for the denaturation model. 19](#_Toc208828303)

[Fig. S11. Proposed reaction mechanisms for the MBH reaction. 20](#_Toc208828304)

[Fig. S12. Computational metrics for de novo designed MBHases. 21](#_Toc208828305)

[Fig. S13. Michaelis-Menten kinetics for MBH designs. 22](#_Toc208828306)

[Fig. S14. HPLC traces for the MBH reaction. 23](#_Toc208828307)

[Fig. S15. Circular dichroism data of MBH48. 24](#_Toc208828308)

[Fig. S16. Electron density for RAD catalytic residues. 25](#_Toc208828309)

[Fig. S17. HPLC analysis for determination of methodol absolute configuration and enantiomeric excess. 26](#_Toc208828310)

[Fig. S18. Backbone Cɑ RMSDs of the simulated RADs for all MD trajectories. 27](#_Toc208828311)

[Fig. S19. ^1^H NMR spectrum of *rac-*methodol 1. 28](#_Toc208828312)

[Fig. S20. ^13^C NMR spectrum of *rac*-methodol 1. 29](#_Toc208828313)

[Fig. S21. ^1^H NMR spectrum of 2-(hydroxy(4-nitrophenyl)methyl)cyclohex-2-en-1-one 5. 30](#_Toc208828314)

[Fig. S22. ^13^C NMR spectrum of 2-(hydroxy(4-nitrophenyl)methyl)cyclohex-2-en-1-one 5. 31](#_Toc208828315)

[Fig. S23. Selection of rotamers for the nucleophilic histidine residue in the MBH reaction. 32](#_Toc208828316)

[Supplementary Tables 33](#_Toc208828317)

[Table S1: Amino acid sequences of RAD constructs. 33](#_Toc208828318)

[Table S2. Mass spectrometry results of purified enzymes. 35](#_Toc208828319)

[Table S3. SAXS parameters. 36](#_Toc208828320)

[Table S4. RAD kinetics, denaturation midpoints, yield, and pKa1 37](#_Toc208828321)

[Table S5. Comparison of designed and evolved Retro-aldol enzymes. 39](#_Toc208828322)

[Table S6. Amino acid sequences of MBH constructs. 40](#_Toc208828323)

[Table S7: Conversion and design parameters for MBH designs 44](#_Toc208828324)

[Table S8. Comparison of designed and evolved MBHases. 46](#_Toc208828325)

[Table S9. Primers used for generation of RAD variants. 47](#_Toc208828326)

[Supplementary References 49](#_Toc208828327)

# Supplementary Methods

## The Riff-Diff pipeline

The full codebase to the latest version of Riff-Diff including detailed instructions for use can be found at <https://github.com/mabr3112/riff_diff_protflow>. This repository will be kept up to date and also includes all Rosetta .xml scripts that are used during the refinement steps of the protocol. The following section is a detailed description of the design steps performed by the scripts of the Riff-Diff pipeline.

***Motif library generation***

Motif library construction can proceed via two modes: (1) using a predefined backbone structure (“backbone rotamer finder”), or (2) extracting fragments from a filtered PDB database (“fragment finder”).

*Backbone rotamer finder*

By default, a 7-residue helical fragment (with phi/psi angles of -64.8/-41.0 at each position) is used as input. For each residue in the catalytic array, the rotamer library of the corresponding amino acid is retrieved. For each position on the fragment that is not on the N- or C-terminus, the phi and psi angles are determined (default -64.8/-41.0) and the corresponding rotamers are extracted from the Dunbrack rotamer library^32^ as provided with Rosetta. Rotamers with <5% overall probability and <30% of the probability of the highest likelihood rotamer at a given fragment position are filtered out. To expand conformational diversity, additional rotamers are generated by systematically perturbing each chi angle by ±1 and ±2 standard deviations from the mean values provided in the rotamer library. The rotamers are ranked according to a composite score of the log rotamer probability and the log phi/psi angle occurrence for the corresponding amino acid identity. The selected rotamers are attached to each specified fragment position. A maximum number of 15 fragments (corresponding to 15 unique rotamers) per input position on the fragment is returned.

*Fragment finder*

For each residue in the catalytic array, the rotamer library of the corresponding amino acid identity is retrieved. The rotamers are ranked according to a composite score of the log rotamer probability and the log phi/psi angle occurrence for the corresponding amino acid identity. If a fragment secondary structure was provided (e.g. helical), the rotamer library is filtered for all phi/psi angles that correspond to the selected secondary structure. Only rotamers meeting all of the following criteria are retained: (i) the backbone phi/psi combination must occur in at least 0.5% compared to all phi/psi combinations for the given amino acid, (ii) the rotamer itself must have a probability of at least 5%, and (iii) its probability must be at least 30% of that of the most probable rotamer. An example for this filtering step is provided in Suppl. Figure S23. The rotamers are ranked according to a composite score of the log rotamer probability and the log phi/psi angle occurrence for the corresponding amino acid identity. A maximum number of 15 rotamers per fragment position is returned.

From a version of the PDB containing structures with a maximum sequence identity of 20%, all positions with the same amino acid identity, identical phi, psi (both rounded to the nearest ten) and chi angles (± two standard deviations) as well as the three adjacent residues in both directions are extracted, creating fragments of 7-residue length. The extracted fragments are filtered for secondary structure content (e.g. 100% helical). All extracted fragments are grouped by the phi/psi/omega angles (rounded to the nearest ten) of all residues in the fragment and assigned a score that corresponds to the number of fragments in each group. This score (referred to as “backbone probability”) indicates how common this fragment is in the PDB. For all fragments within a group, the median of phi/psi/omega is calculated and an updated fragment is generated using these values. The resulting fragments are ranked according to their log backbone probability, log rotamer probability and log phi/psi occurrence.

*Motif library*

The selected fragments are superimposed with the input catalytic array via their functional groups. A clash detection is performed, removing all fragments that show clashes with the ligand. Additionally, fragments for each residue in the catalytic array are filtered for a minimal backbone RMSD (0.3 Å) to all other selected fragments. Next, clash detection between the fragments of each residue in the catalytic array is performed. All clashing fragment combinations are removed. The resulting motifs are ranked according to their average backbone probability (for fragments that are derived from Fragment Finder protocol), rotamer probability and phi/psi occurrence. The placeholder helix is added in the last step to create the final catalytic motifs.

***Structure generation***

This section describes the Riff-Diff pipeline as used for the generation of MBH constructs with default settings. The original version that was used to design RAD constructs is largely similar, but used a ProteinMPNN-informed Rosetta FastDesign for sequence generation instead of LigandMPNN. To sample efficiently, FastDesign could only exchange amino acids within the active site and was constrained at each position to residue identities that were assigned high probabilities by ProteinMPNN^69^. The updated version also includes more stringent filtering steps to reduce computational costs. The Riff-Diff structure generation script is divided into 4 phases: Screening, Refinement, Evaluation, and Variant Generation. We refer to design models as “poses” throughout the design trajectory. Unless noted otherwise, RMSDs are calculated relative to orientations in the input catalytic motif and TM scores are calculated between poses and predicted structures. In all LigandMPNN runs, the specified catalytic residues are kept fixed, all other positions are set to designable. After each prediction step (ESMFold or AlphaFold2), the ligand is copied back into the pose via a superimposition of the input motif residues.

*Screening*

The top 200 catalytic motifs are selected as input for structure generation. Per input motif, 5 RFdiffusion trajectories are performed, using 30-residue stretches on the N- and C-terminus and 10-52 residue stretches in between fragments for four 7-residue fragments (example contig: [Q1-21/0 30/A1-7/10-52/B1-7/10-52/C1-7/10-52/D1-7/30], where Q indicates the 21-residue placeholder helix, and A, B, C and D indicate 7-residue fragments). The total length of diffused backbones is exactly 200 residues.

After RFdiffusion, the placeholder helix is removed and the ligand is copied back in, as described above. The poses are filtered based on radius of gyration (< 18 Å, ROG), number of CA-atoms per ligand atom within 8 Å (> 5, referred to as “ligand contacts”), backbone RMSD of the catalytic residues (< 1 Å, referred to as “catres bb RMSD”) and backbone RMSD of all residues in the input motif (< 1 Å, referred to as “motif RMSD”).

LigandMPNN is used to generate initial sequences on all poses that passed the initial filtering (3 per input structure). The returned sequences are threaded onto the input structures and relaxed once with Rosetta, while constraining the catalytic residue side chains as well as the motif backbone atoms to the input motif coordinates. This step will be referred to as “constrained relax” in subsequent paragraphs. Next, sequences are generated based on the relaxed output using LigandMPNN (30 per structure). The resulting sequences are filtered based on a composite score composed of the Rosetta constrained relax total score, ligand contacts after constrained relax, the number of ligand clashes with backbone atoms after constrained relax (referred to as “ligand clashes”, ROG, and the overall confidence score of LigandMPNN. The top 5000 poses are predicted with ESMFold. We chose ESMFold to predict structures throughout screening and refinement cycles because of its faster inference compared to AlphaFold2. Output poses are filtered based on ROG (< 18 Å), motif RMSD (< 1.5 Å), catres bb RMSD (< 1.5 Å), TM score to the design model (> 0.9), average per-residue ESMFold pLDDT (> 70), and ligand contacts (> 5). All passing poses are ranked according to a composite score composed of average per-residue ESMFold pLDDT, TM score, catres bb RMSD, motif RMSD, ligand clashes, ligand contacts, and ROG. The top scoring pose according to these metrics for each RFdiffusion output is kept for the next phase.

*Refinement*

For each refinement cycle, poses are constrained-relaxed and 25 sequences are generated using LigandMPNN. Next, the design sequences are predicted using ESMFold and filtered based on catres bb RMSD, motif RMSD, average ESMFold pLDDT, ligand contacts (> 5) and TM score (> 0.9). Filter cutoffs for catres bb RMSD, motif RMSD and average pLDDT are ramped with increasing number of refinement cycles, starting at 1.2 Å/ 1.5 Å/ 75 and ending at 0.7 Å / 1.0 Å / 85. All passing poses are ranked according to a composite score (referred to as “ref comp score”) composed of these scoreterms and RMSD of catalytic residue side chains (referred to as “side chain RMSD”), and ligand clashes. The top three poses per unique RFdiffusion output are passed to the next refinement cycle. After five cycles of refinement, the top 500 poses according to ref comp score are passed on for evaluation.

*Evaluation*

For each designed sequence, five predictions using AlphaFold2 in single sequence mode are performed. The top pose according to average pLDDT is selected, and poses are filtered according to average pLDDT (> 85), TM score (> 0.9), catres bb RMSD (< 0.7 Å), and ligand contacts (> 5). Poses (without ligand) are repacked using AttnPacker^70^ and side chain RMSD is calculated. All passing poses are relaxed with the ligand present (without constraints) 15 times, and the mean sidechain RMSD, ligand RMSD, and catres bb RMSD are calculated. The poses are ranked according to the evaluation composite score (all of above mentioned values and the number of ligand clashes and surface aggregation propensity (SAP)).

*Variant generation*

In the final step of the Riff-Diff pipeline, custom mutations can be introduced upon inspection of the evaluation poses. This is useful e.g. to open channels that are blocked by residue side chains.

Poses are relaxed with constraints. If mutations were specified, the amino acid identities at the specified positions are restrained during the following LigandMPNN step (50 sequences per pose). The resulting sequences are predicted using ESMFold and filtered in the same way as the final refinement step. All passing sequences are predicted again with AlphaFold2 and filtered and ranked in the same way as during the evaluation step.

Instead of LigandMPNN-based variant generation, an alternative Rosetta coupled-moves based protocol can be employed. Following constrained relax, this protocol is performed 50 times for each pose. Designable positions are selected based on the DetectProteinLigandInterface TaskOperation with cut parameters of 6, 8, 10 and 12 Å. For each designable position, the occurrence of each amino acid in the coupled moves output sequences is evaluated. Amino acid identities chosen in 25% of all trajectories (or all above 10%, if none passes this cutoff) are selected, and every possible combination of residue identities is generated. The resulting sequences are then predicted with ESMFold and AlphaFold2, as described above.

***Implementation of the placeholder helix and a custom auxiliary potential to scaffold substrate pockets***

The artificial motifs introduced in the Riff-Diff pipeline contain an additional helix that is used as a placeholder for a substrate pocket during structure generation with RFdiffusion. This helix can be placed manually or in an automated manner into the artificial motif library. With manual placement, the user places the helix directly into the initial catalytic array, which can then be identified by the scripts that generate the artificial motif library. The automatic placement mode calculates a vector *V* between the center of mass of (a) the atoms of the artificial motif and (b) the substrate atoms. The first alpha carbon of the helix is then placed on the center of mass of the substrate atoms and oriented along vector *V* to point away from the motif. In general, any arbitrary backbone can be used as a placeholder. The automatic placement mode uses a straight alpha-helical fragment of 21 residues with a sequence starting with small side chains (Gly) that increase in size to mimic a cone shape. The sole function of the placeholder helix is to provide a negative shape into which RFdiffusion cannot scaffold a backbone.

Without auxiliary potentials, RFdiffusion generated extended scaffolds that pointed away from the placeholder helix. To ensure that the scaffolds generated by RFdiffusion were both globular and centered on the artificial motif and its channel placeholder, we implemented a custom auxiliary potential. The auxiliary potential implements a radius of gyration loss to a virtual center of mass. This virtual center of mass is calculated using a recentering vector scaled by a customizable *distance* factor. Similar to the placement of the placeholder helix, the user can either specify the direction of the recentering vector ***v*** using xyz coordinates or use this potential in an automated mode. The automated mode calculates the vector *v̂* as the vector from the substrate atom centroid to the channel helix centroid. Equation (1) describes this potential, where *w* corresponds to the weight of the auxiliary potential, *d* to the distance factor of the recentering, *v̂* to the normalized vector, *r_i_* to the coordinates of every C_ɑ_ atom, and *N* to the number of C_ɑ_ atoms in the structure.

$P_{aux}= -w \sqrt{\frac{1}{N}\sum_{i=1}^{N} (r_{i}-d\hat{v})^{2}}\cdot N^{1/3}$ (1)

## Prediction of stability of designed retro-aldolases

To predict resistance to chemical denaturation, we fit a linear regression model on a composite score consisting of four in-silico metrics: AlphaFold2 pLDDT and three Rosetta-derived metrics – total_score, an atomic contacts score (AtomicContactCount filter of RosettaScripts), and surface aggregation propensity (SAP). Each Rosetta metric was normalized by sequence length of the designed enzymes to remove size-dependence. The composite score of the four metrics was calculated after normalizing each metric to zero median, unit variance, and multiplication by the sign of the metric’s correlation with the experimentally determined GdnHCl melting point. The formula to calculate this composite score is shown in equation (2), where *s_i_* corresponds to the sign of the correlation, and *x̄_i_* and *σ_i_* to the median and variance of the corresponding metric. We then fit slope (β_1_) and intercept (β_0_) of the model using ordinary least squares fitting (equation 3) using the python library SciPy, version 1.13.1. Leave-one-out cross-validation (LOOCV) demonstrated that the model explains 59% of the observed variance in denaturation midpoint. The regression coefficients and scaling factors for the four computational metrics are reported in equation (4). We note that the predictive power of this model is likely limited to folds designed by the Riff-Diff pipeline.

$\underline{T}=\sum_{i} s_{i}\cdot(\frac{x_{i}-\underline{x}_{i}}{\sigma_{i}})$ (2)

$y=\beta_{1}\cdot\underline{T}+\beta_{0}$ (3)

$$GdnHCl_{pred} = 0.318 \cdot(\frac{pLDDT-93.7}{1.43} +\frac{contacts - 3.08}{0.363}-\frac{totalscore + 2.69}{0.0803}+\frac{SAP-0.434}{0.0901})+3.78 (4)$$

## AlphaFold3 active site metric

The active site metric combines interaction geometries of two reaction states, the michaelis complex and the hemiaminal intermediate. In the michaelis complex, we evaluated the attacking conformation of the nucleophilic lysine towards the R-methodol keto-group using three metrics: the distance between the lysine NZ to the ketone, the angle spanned by the lysine NZ atom, the carbonyl carbon and oxygen, and the dihedral angle between the lysine NZ atom, the carbonyl carbon, carbonyl oxygen and the first carbon substituent of the keto-group. To evaluate the interactions with the covalent hemiaminal intermediate, we measured four interatomic distances: Y51-OH, Y180-OH and N110-N to the hemiaminal hydroxy-group and Y51 to the beta hydroxy-group. For each design and RA95.5-8F, 20 predictions were generated with AlphaFold3^71^ using random seeds.

To facilitate addition of the individual measurements of both states, each measurement of an interaction geometry was normalized by the corresponding measurement in the reference predictions (RA95.5-8F). In this normalization procedure, we subtracted the median of the corresponding measurement *x̄_ref_* in RA95.5-8F and normalized the difference by its median absolute deviation *MAD_re_*_f_. We chose median absolute deviation as the normalization factor because it is more stable to outliers than standard-deviation. Finally, we average the interaction geometries within each reaction state for each prediction. The full normalization procedure for the interaction geometries of a reaction state prediction is shown in equation 5. The active site metric *m_AS_* is calculated by selecting and summing the predictions with the lowest score of each reaction state (equation 6). Thereby the active site metric combines the predicted interactions of both reaction states, normalized by the median absolute deviation of the interaction in the reference prediction.

$d=\sum_{n} (\frac{x_{n}-\underline{x}_{n,ref}}{{MAD}_{n,ref}})\div n$ (5)

$m_{AS} =\min_{n=20}(d_{ketone})+\min_{n=20}(d_{hemi})$ (6)

## Design of MBH constructs

***Creation of catalytic arrays***

The geometry of His23 and Arg124 as well as the substrates’ orientations were extracted from a previously reported density-functional theory (DFT) model of transition state 1 from the BH32.14-catalyzed reaction of cyclohexenone (**3**) with 4-nitrobenzaldehyde (**4**). These geometries were then used as input for the Riff-Diff pipeline. As the reported essential residue Glu46 was not present in the DFT model, we modeled a carboxylic acid interacting with the histidine Nδ-hydrogen. An additional Trp side chain forming π–π-stacking interactions with **4** was also added to facilitate better substrate binding. We used geometry optimization with XTB^72^, version 6.6.1, with the generic force field GFN-FF using the ALPB implicit water solvent model to calculate the interaction between imidazole (histidine side chain) and acetic acid (aspartic and glutamic acid side chains). The orientation of the tryptophan side chain relative to 4-nitrobenzaldehyde was modeled in the same way. Subsequently, we used CREST^73^ version 2.12 with the GFN-2 method and ALPB implicit water solvent model to identify the lowest-energy conformers of the tryptophan side chain. The carboxylic acid and tryptophan orientations were combined with the original active site models to create the input for the Riff-Diff pipeline.

For designs modeled after the active site of BH1.8, we extracted the coordinates of the Nδ-methylhistidine, Glu26 and the substrates from transition state 4 (Supplementary Figure S11b) of quantum mechanics/molecular mechanics (QM/MM) calculations of the BHMeHis1.8-catalyzed reaction of 3 with 4 and combined them with the previously generated carboxylic acid and tryptophan orientations to create a catalytic array (Figure 5c).

***Riff-Diff Design***

For 36 of the designs based on the active site of BH1.8, the helical fragment used for attachment of the active site residue was not predetermined (Backbone rotamer finder). Instead, the Fragment finder approach to motif library generation was employed, selecting only fragments with 100% helical secondary structure according to DSSP^71^. After screening, refinement and evaluation with AlphaFold2, 7 of these constructs were further refined with the original coupled moves protocol. All other 38 designs were refined with LigandMPNN. A complete list of constructs, the design method and substrate conversion can be found in Supplementary Table S7. To select sequences based on the active site of BH1.8, we included the glutamic acid pKa calculated with Propka^74^ and the ligand heavy atom RMSD between the design model and AlphaFold3 predictions with the product in our evaluation metrics.

## Fitting of pKa values for RAD constructs

pKa values were obtained according to a fit with a two-pKa model (equation 7) using the Python library SciPy, version 1.15.2.

$\frac{k_{cat}}{K_{M}}=\frac{C}{1 + {10}^{(pKa,1 -pH)} - {10}^{(pH - pKa,2)}}$ (7)

*k*_cat_ and *K*_M_ have their usual meaning, C corresponds to the maximum value of *k*_cat_ / *K*_M_, and pKa_1_ and pKa_2_ are the logarithmic acid dissociation constants.

## Substrate inhibition

Michaelis Menten kinetics of RAD29 variants were fitted using the Haldane substrate inhibition model (equation 8) using the python library SciPy, version 1.13.1. Inhibition constants are reported as K_i_ in the corresponding panels (Supplementary Figure S7).

$v= \frac{V_{m}[S]}{K_{M}+[S]+\frac{{[S]}^{2}}{K_{I}})}$ (8)

*K*_M_ has its usual meaning, *V*_m_ is the maximum reaction rate, [S] denotes substrate concentration and *K*_I_ is the inhibition constant.

## Fitting of Michaelis-Menten kinetics for MBH constructs

Initial reaction velocities (V_0_) at each substrate concentration were determined from linear fits of conversion versus time. The combined V_0_ versus **3** and V_0_ versus **4** were fitted globally using a random order binding model (equation 9):

$v= \frac{k_{cat}[E][A][B]}{{(K}_{M,A}+[A])(K_{M,B}+[B])}$ (9)

where *k*_cat_ is the catalytic constant, [E] is the total enzyme concentration, [A] and [B] are the initial **3** and **4** concentrations, respectively, and *K*_M,A_ and *K*_M,B_ are the corresponding apparent Michaelis constants. Fits were performed with shared *k*_cat_, *K*_M,A_ and *K*_M,B_ values as a three-dimensional surface fit using the Python library SciPy, version 1.15.2. Fits for individual measurements versus **3** and **4** are shown in Supplementary Figure S13.

# Supplementary figures


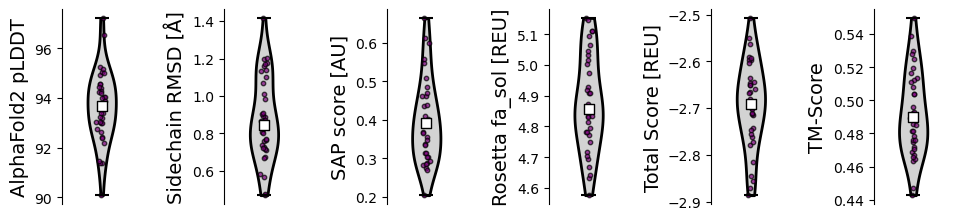


## Fig. S1. Computational metrics of selected RAD models.

All 36 sequences were assigned high confidence by AlphaFold2. The displayed AlphaFold2 pLDDT corresponds to the average per-residue pLDDT per structure. The average sidechain RMSD to the catalytic geometry was below one angstrom. SAP and Rosetta scores were calculated per residue. The displayed TM scores represent the TM score calculated by TMAlign to the closest match in the PDB by Foldseek. White squares correspond to the median of the distributions, purple dots to individual designs.


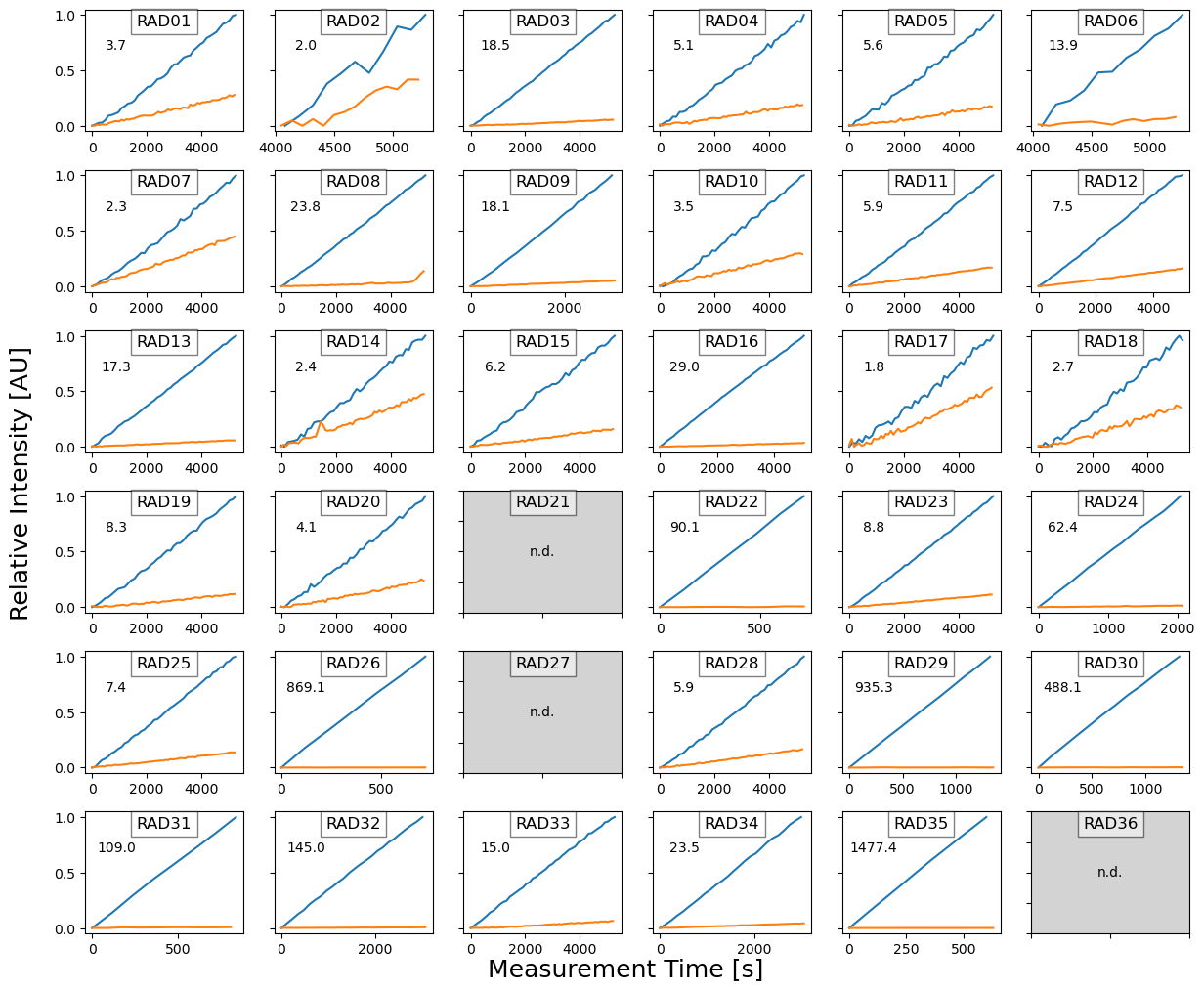


## Fig. S2. Mutation of active site lysine residues to alanine leads to significant loss of activity of designed retro-aldolases.

Relative reaction progress for original constructs (blue) and active site lysine to alanine variants (orange). Numbers denote n-fold decrease in activity. Variants of highly active constructs RAD29 and RAD35 display 935.3 and 1477.4-fold decrease, respectively.


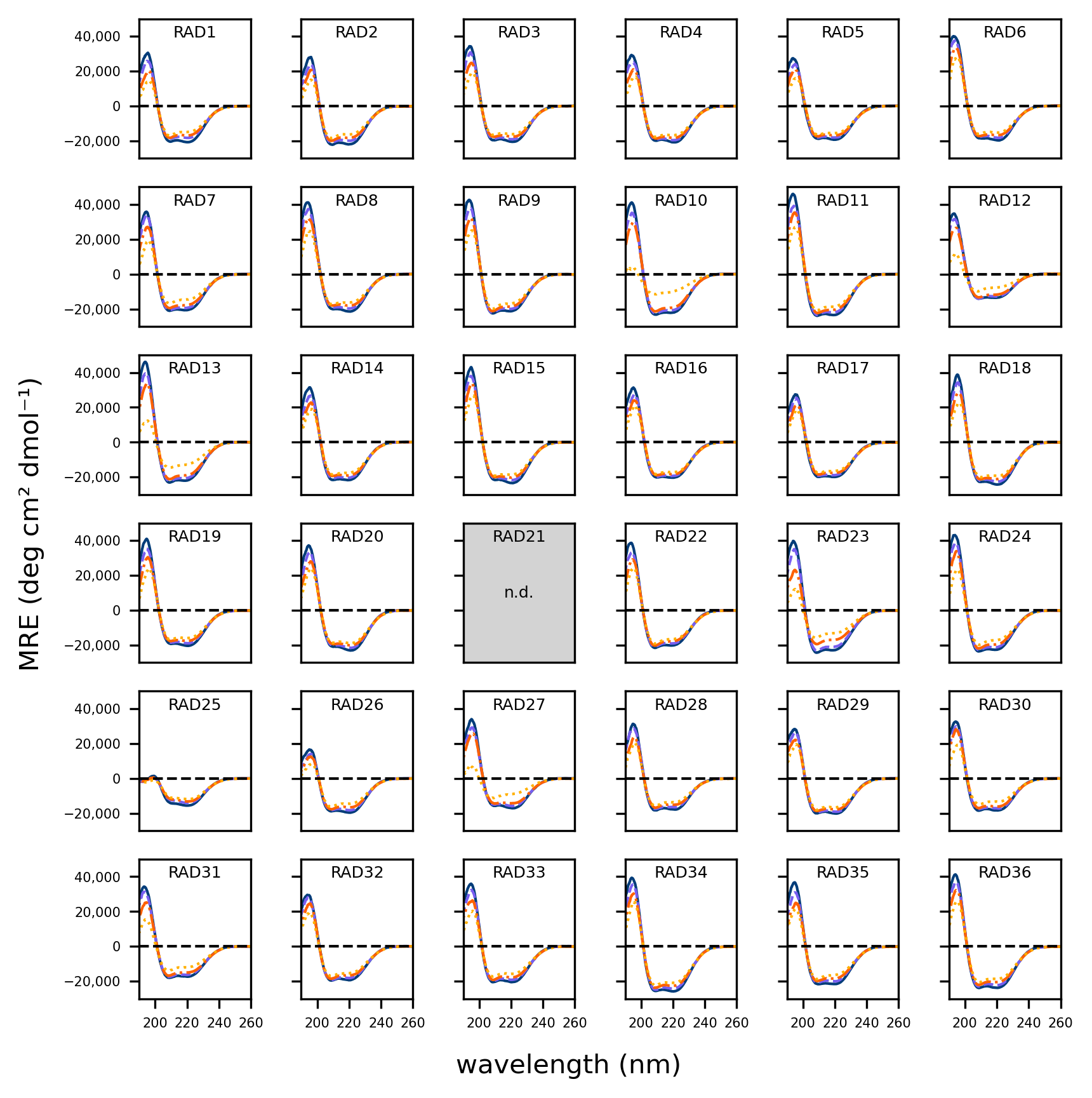


## Fig. S3. CD spectra of designed RAD enzymes.

Circular dichroism spectra of designed RAD enzymes at 20 °C (solid blue), 45 °C (dashed purple), 70 °C (dashed dotted orange) and 95 °C (dotted yellow) confirm alpha-helical fold and high thermodynamic stability. For 30 constructs, heating the samples to 95 °C yielded a decrease in CD signal intensity at 220 nm between 75% to 85% of the initial value, indicating only marginal unfolding (Figure 3a). RAD10, RAD12, RAD13, RAD27 and RAD31 show the onset of unfolding at 95°. The thermal denaturation curve for RAD23 shows a higher degree of unfolding compared to all other constructs starting at 60 °C but no clearly defined melting temperature can be assigned, indicating uncooperative unfolding. Except for RAD10 and RAD13, the CD spectra after heating to 95 °C and cooling to 20 °C closely resemble the spectra before the thermal scan was performed.


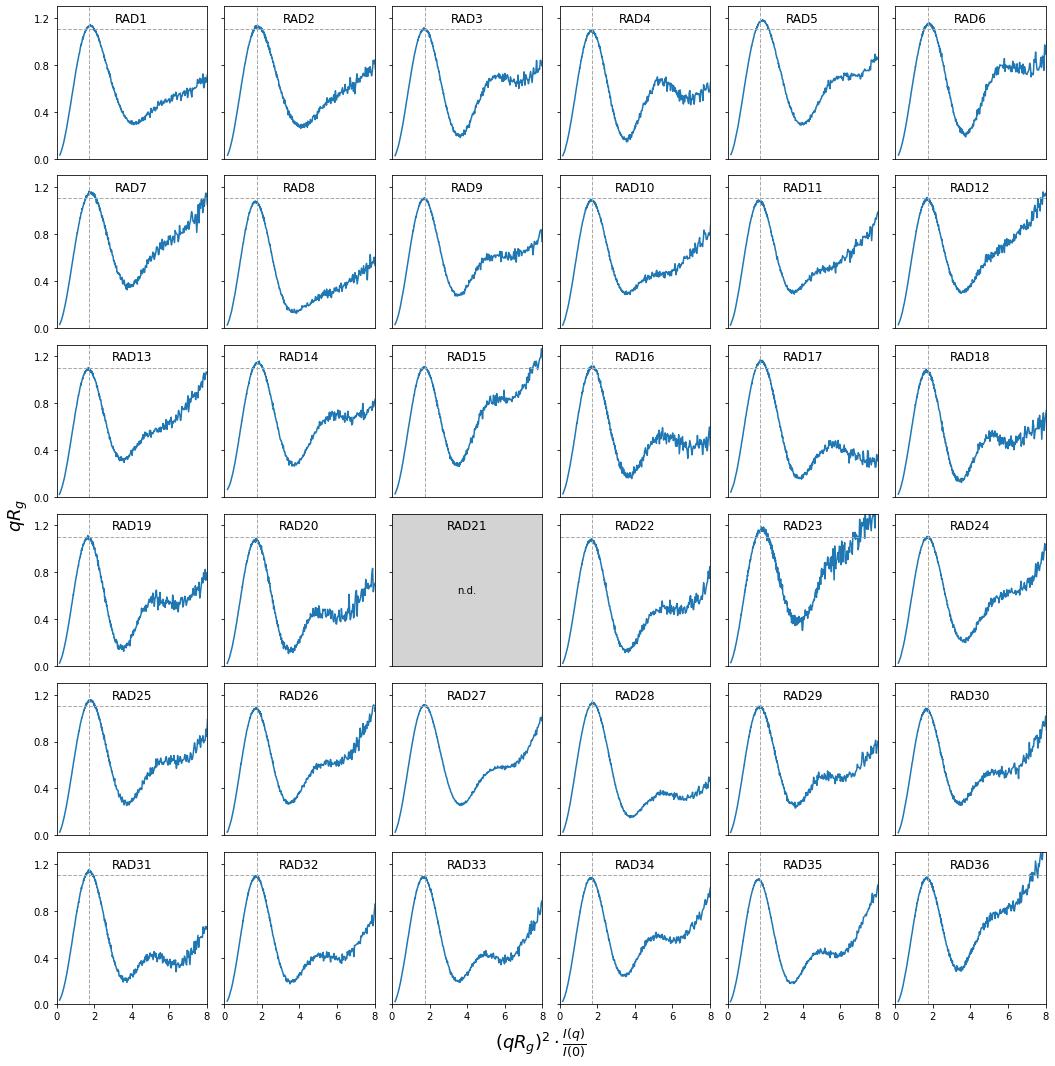


## Fig. S4. Dimensionless Kratky plots

Dimensionless Kratky plots derived from experimental SAXS profiles confirm globular shape and high degree of foldedness of the designed enzymes, indicated by the peak maxima close to Rg = 3^1/2^ and (qR_g_)^2^I(q)/I(0) = 1.104 (dotted lines).


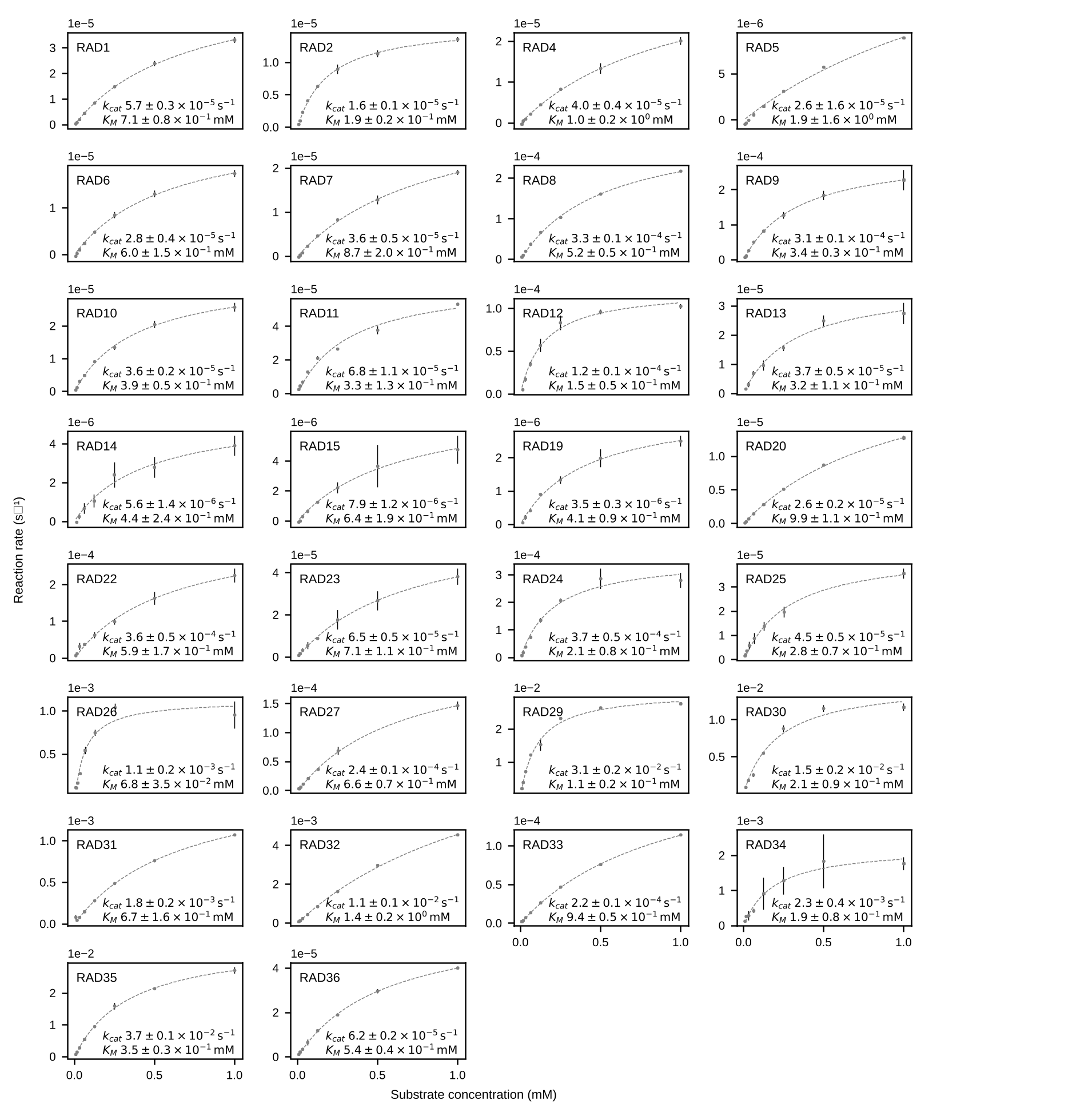


## Fig. S5. Michaelis Menten kinetics of RADs

Michaelis Menten kinetics of (**a**) designed retro-aldolases with *rac*-methodol. Reaction rates at individual concentrations were measured in triplicates. Error bars indicate standard deviations. Designs for which Michaelis Menten parameters could not be obtained were omitted.


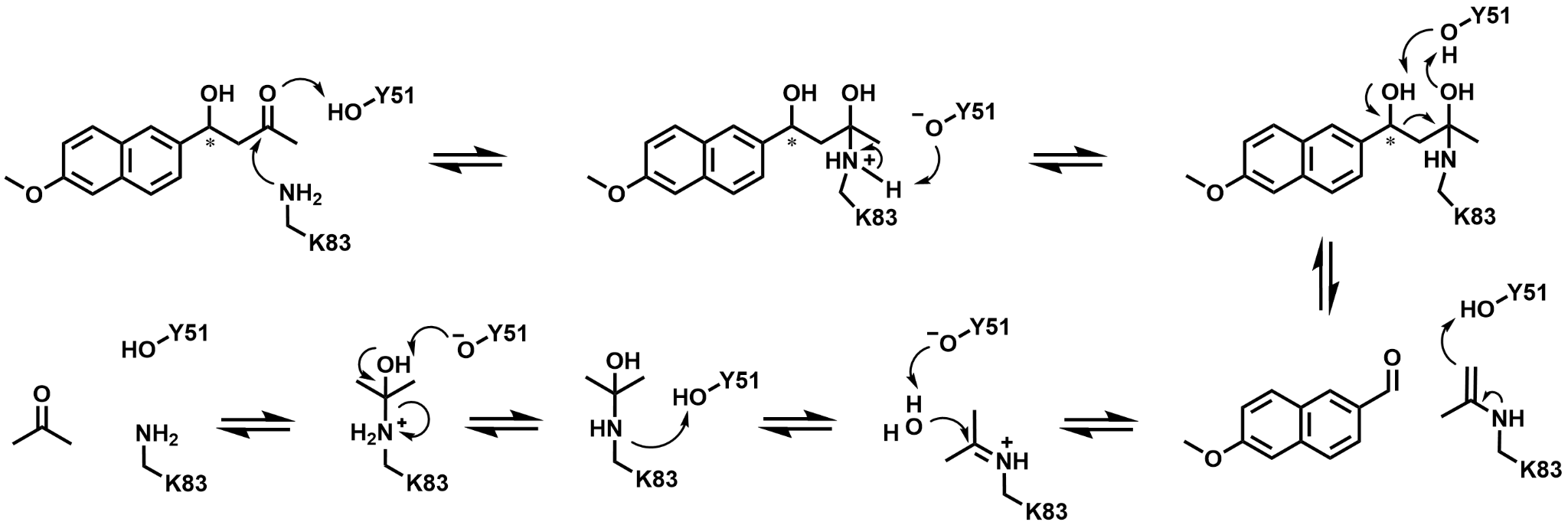


## Fig. S6. Proposed reaction mechanism for the RA98.5-8F-catalyzed retro-aldol reaction of methodol.


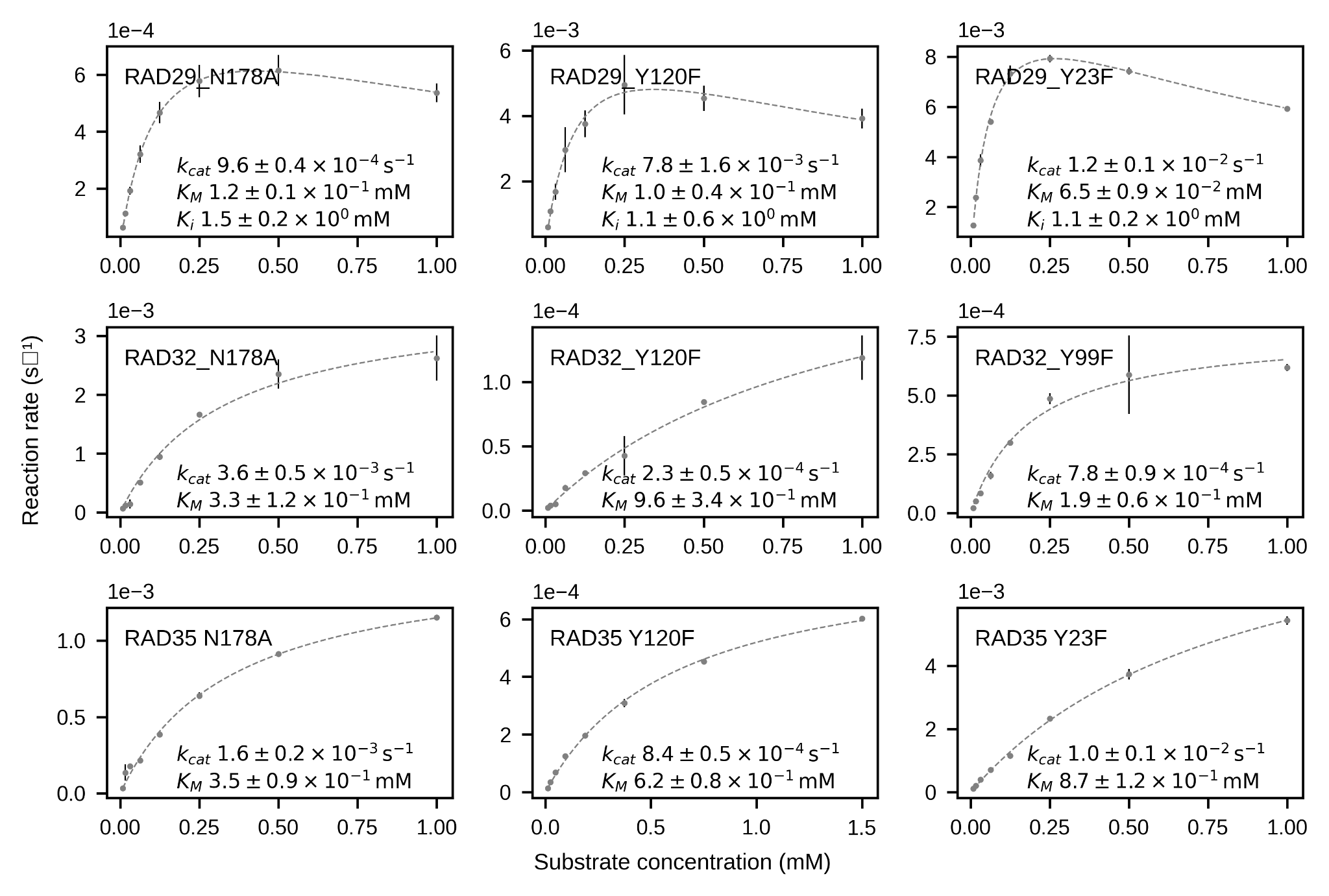


## Fig. S7. Michaelis-Menten kinetics of RAD variants

Michaelis Menten kinetics of site-directed mutagenesis variants of selected retro-aldolases with *rac*-methodol as substrate. Reaction rates at individual concentrations were measured in triplicates. Error bars indicate standard deviations of triplicate measurements. RAD29 variants were fitted using the Haldane substrate inhibition model (equation 8) and the inhibition constants are reported as *K*_i_ in the corresponding panels.


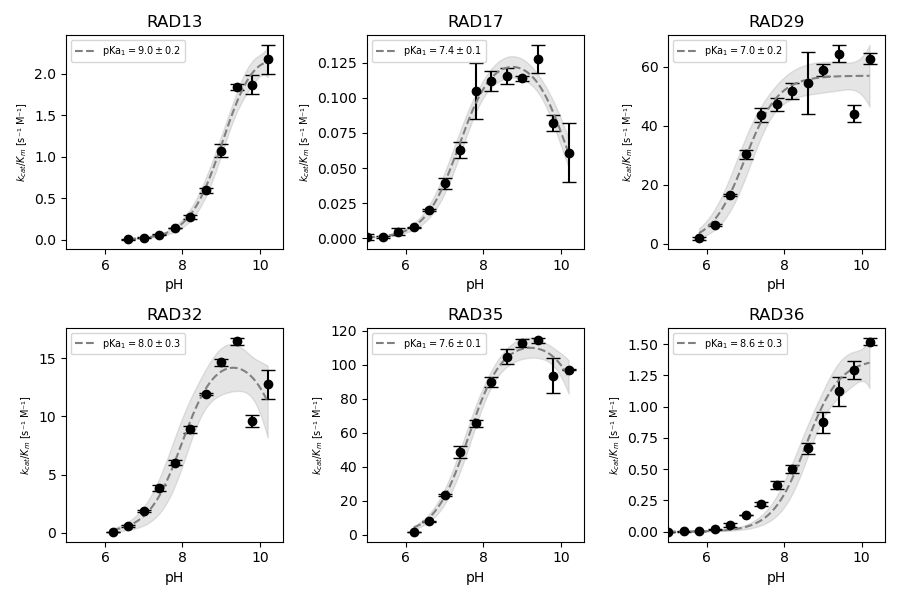


## Fig. S8. pH profiles of RADs.

Gray areas indicate the 95% confidence interval of the fit, error bars indicate standard deviations of triplicate measurements.


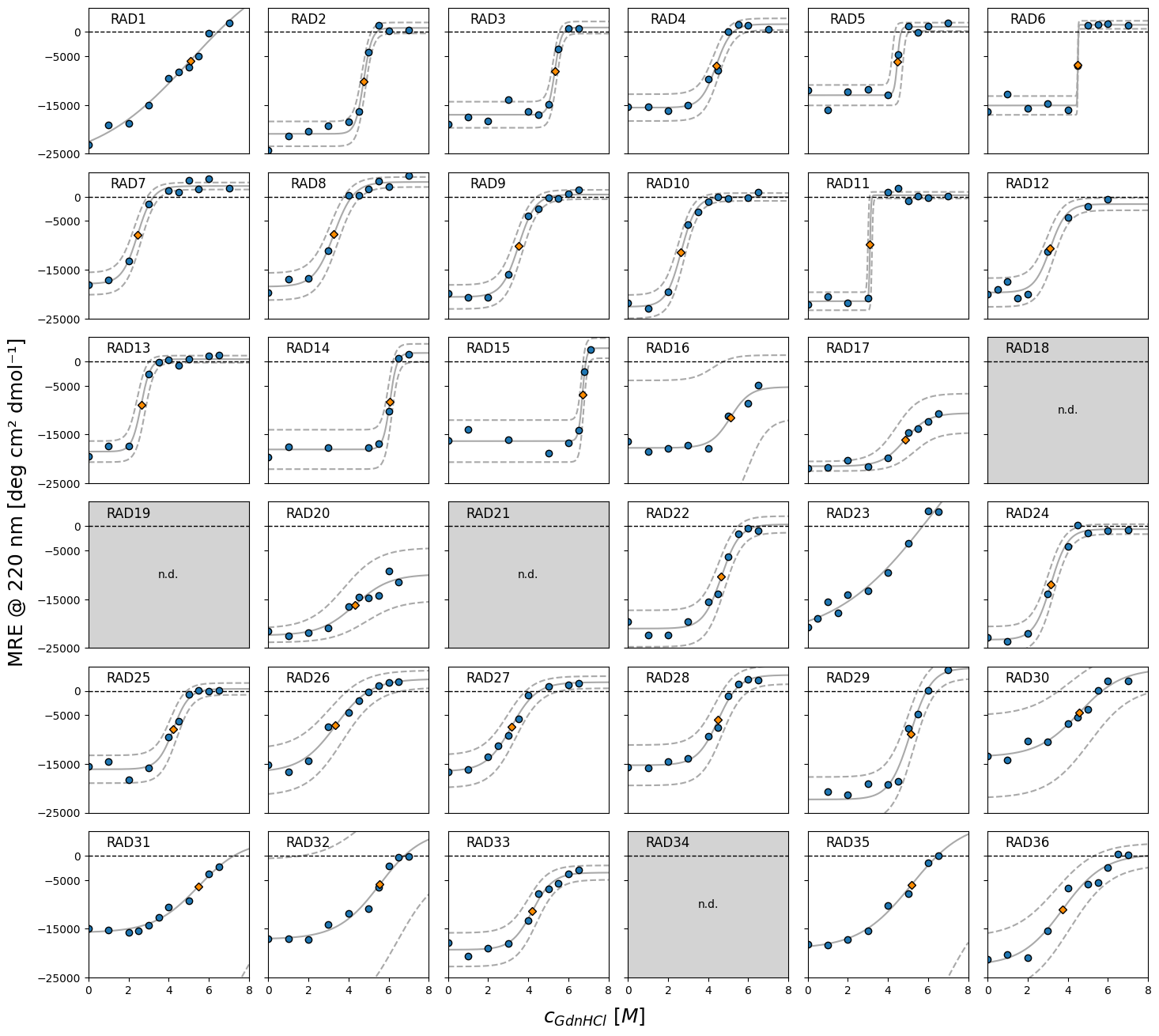


## Fig. S9. Chemical denaturation of RAD designs

Chemical denaturation with GdnHCl monitored via CD spectroscopy at 220 nm (MRE = mean residue ellipticity). Orange dots represent the denaturation midpoints calculated from a sigmoidal fit (gray lines). The confidence interval (95%) of the fit is shown in dotted gray lines. Highest resistance to chemical denaturation was observed for RADs 14-17 and RAD20, with midpoints of the denaturation transition above 6 M of guanidinium hydrochloride (GdnHCl). RAD1, RAD23, RAD30, RAD31, RAD32 and RAD36 displayed non-cooperative unfolding, impeding determination of accurate denaturation midpoints. RAD18, RAD19 and RAD34 were not measured. All other constructs display denaturation midpoints between 2.6 and 5.3 M GdnHCl.

**
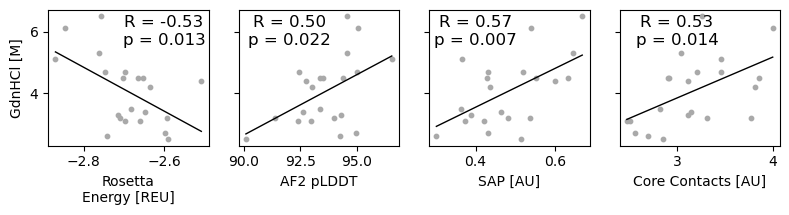
**

## Fig. S10. Individual correlations of computational metrics used for the denaturation model.

Correlations and statistical significance were calculated using python libraries numpy and SciPy. AF2 pLDDT corresponds to the average per-residue pLDDT per structure of AlphaFold2 predictions. Rosetta scores and SAP score were calculated per-residue as metrics to be independent of the protein size. Core contacts were calculated using the RosettaScripts AtomicContactCount filter and represent the atomic density of the protein.


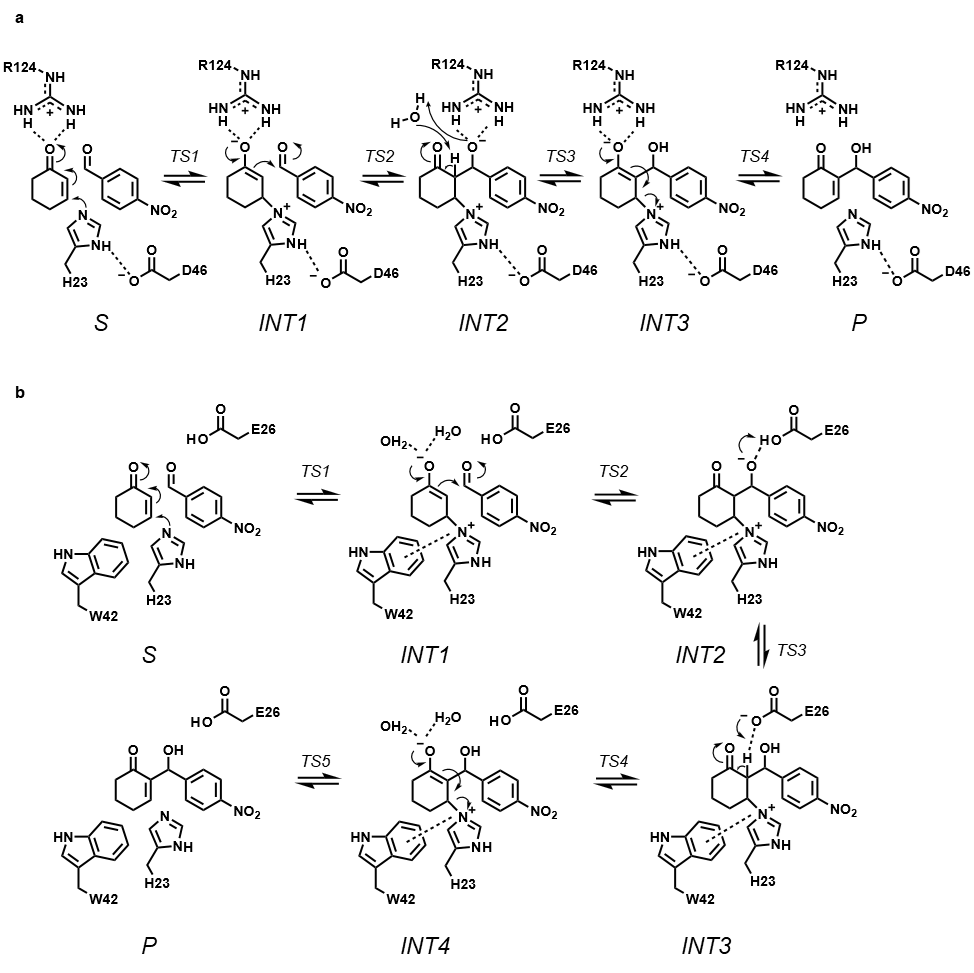


## Fig. S11. Proposed reaction mechanisms for the MBH reaction.

Proposed reaction mechanism for the BH32.14-catalyzed (**a**) and BH1.8 23H-catalyzed (**b**) Morita-Baylis-Hillman reaction of 2-cyclohexen-1-one (**3**) and 4-nitrobenzaldehyde (**4**). In the original publication of the BH1.8 mechanism, Int3 was called Int2H and transition state numbers were assigned differently compared to this study^30^.


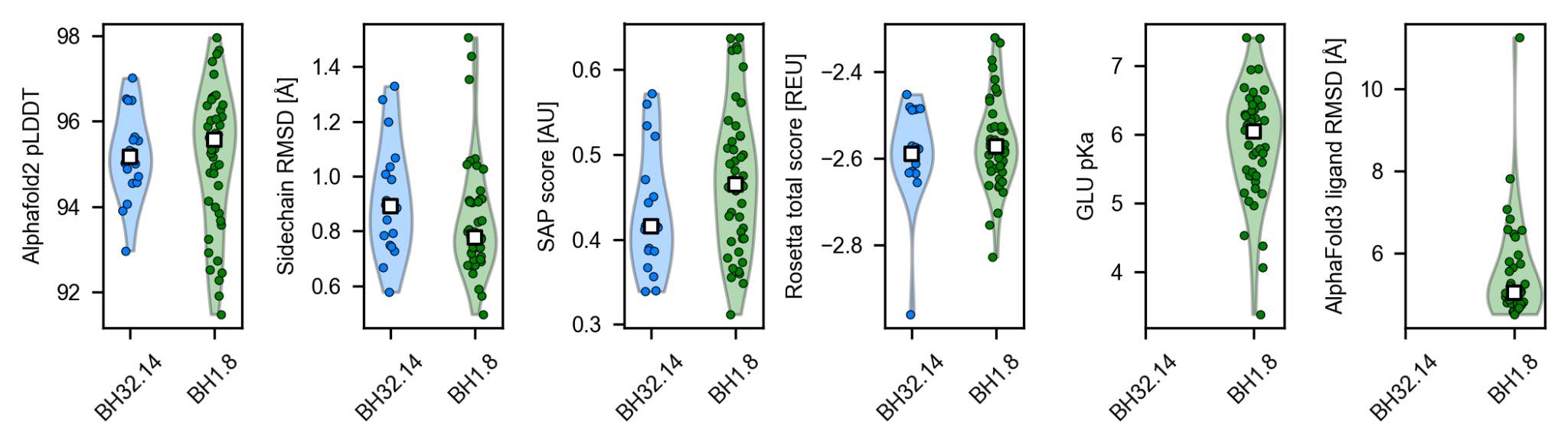


## Fig. S12. Computational metrics for de novo designed MBHases.

Computational metrics for de novo designed MBHases for each set of active site models. White squares indicate median values, dots indicate values of individual designs. Alphafold2 pLDDT corresponds to the average per-residue pLDDT per structure. Sidechain RMSD was calculated from AttnPacker-repacked AlphaFold2 predictions compared to the geometry of the catalytic array. Surface aggregation propensity (SAP) and Rosetta total energy are divided by the number of residues in each design. For designs based on the active site of BH1.8, the PROPKA-calculated pKa of the glutamic acid residue facilitating the proton transfer and the RMSD of the MBH reaction product compared to the substrate in the catalytic array is provided as well.


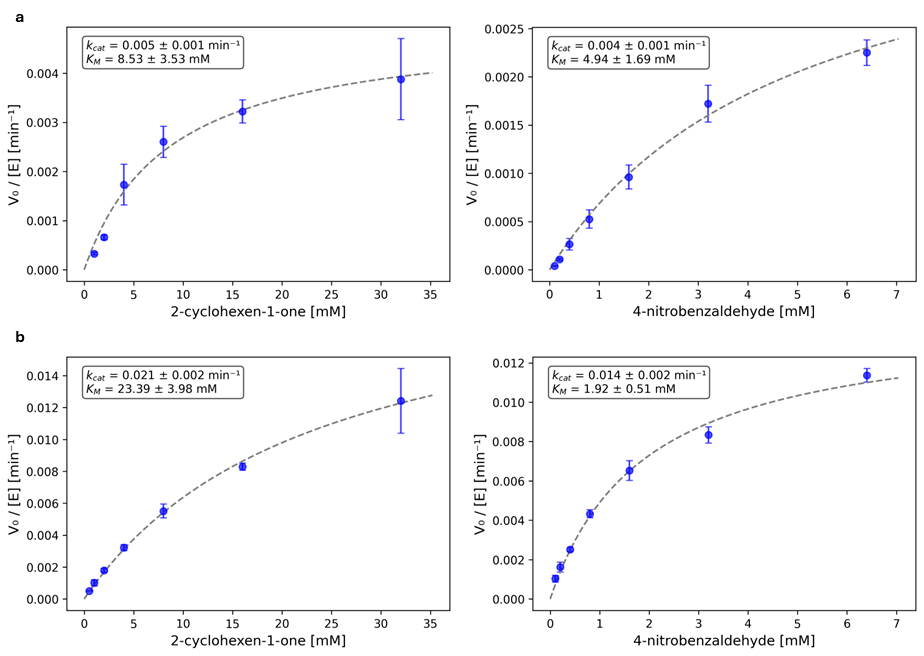


## Fig. S13. Michaelis-Menten kinetics for MBH designs.

Fits of initial velocities against 2-cyclohexen-1-one (**3**) and 4-nitrobenzaldehyde (**4**) for MBH18 (**a**) and MBH48 (**b**). Error bars indicate standard deviations of triplicate measurements.

**
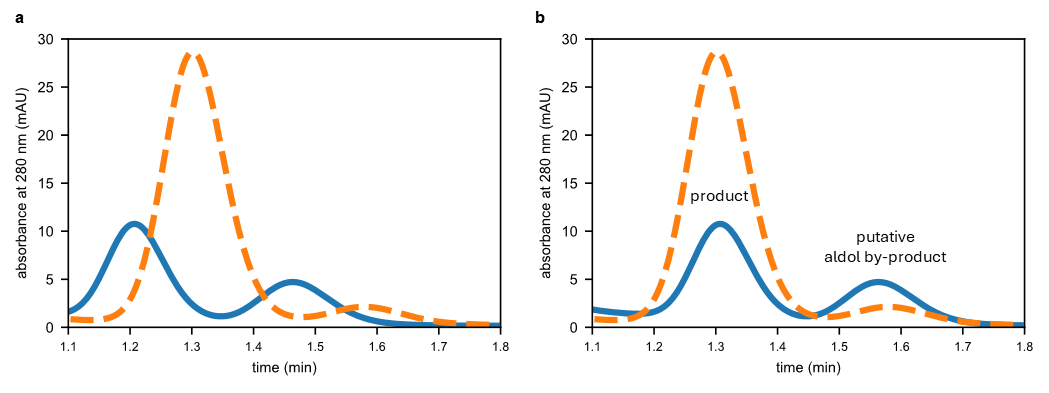
**

## Fig. S14. HPLC traces for the MBH reaction.

**HPLC traces for the MBH reaction of 2-cyclohexen-1-one (3) and 4-nitrobenzaldehyde** (**4**) **catalyzed by MBH18 (solid blue) and MBH48 (dashed orange).** Panel **a** shows the original HPLC profiles. In panel **b**, the HPLC trace of MBH18 was shifted by 0.1 minutes as we observed a reduction in retention times with increasing number of runs on the HPLC column. The peak on the right shows similar retention times as previously reported for an aldol by-product in the MBH reaction^4,30^.


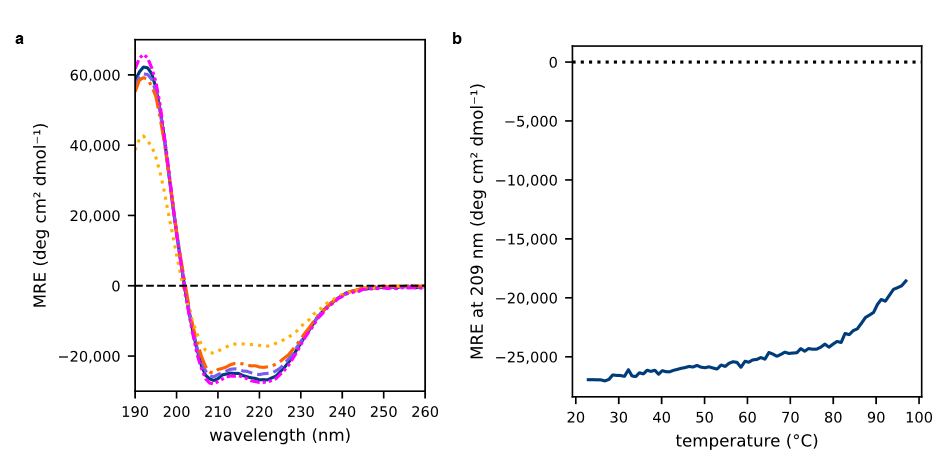


## Fig. S15. Circular dichroism data of MBH48.

**a**, CD spectra at 25 (solid blue), 45 (dashed purple), 70 (dashed-dotted orange), 95 °C (dotted yellow) and after cooling back down to 25 °C (dashed-doubledotted magenta) confirm helical fold. **b**, CD signal intensity at 209 nm versus temperature indicates onset of unfolding at ~85 °C.


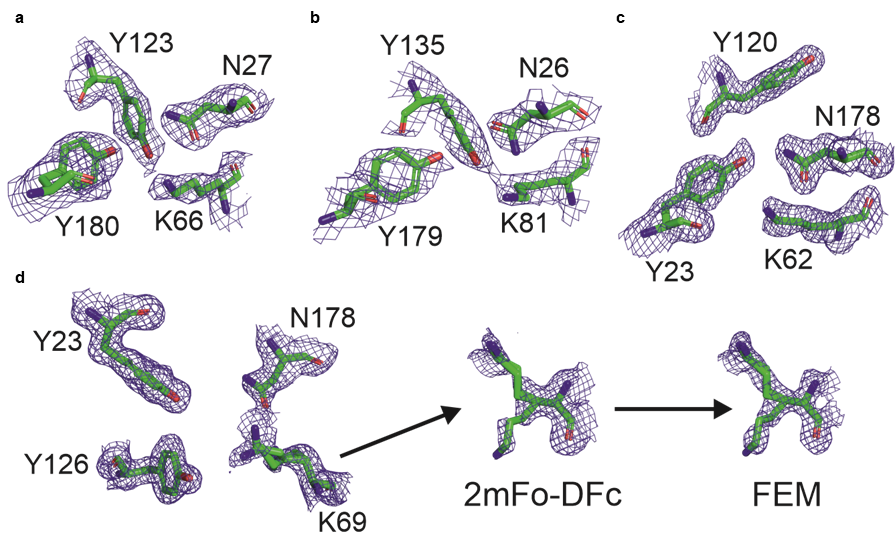


## Fig. S16. Electron density for RAD catalytic residues.

Electron density for the catalytic residues of RAD13 (**a**), RAD17 (**b**), RAD32 (**c**), and RAD36 (**d**). The 2mFo-DFc electron density map contoured at 1.0σ is shown for the tetrad residues of each structurally solved RAD. As residue K69 of RAD36 showed weak and diffuse electron density for multiple conformations (2mFo-DFc), a feature-enhanced map (FEM) was created to improve the features of the conformers. Panel **d** highlights the improvement of the electron density for K69 by FEM.


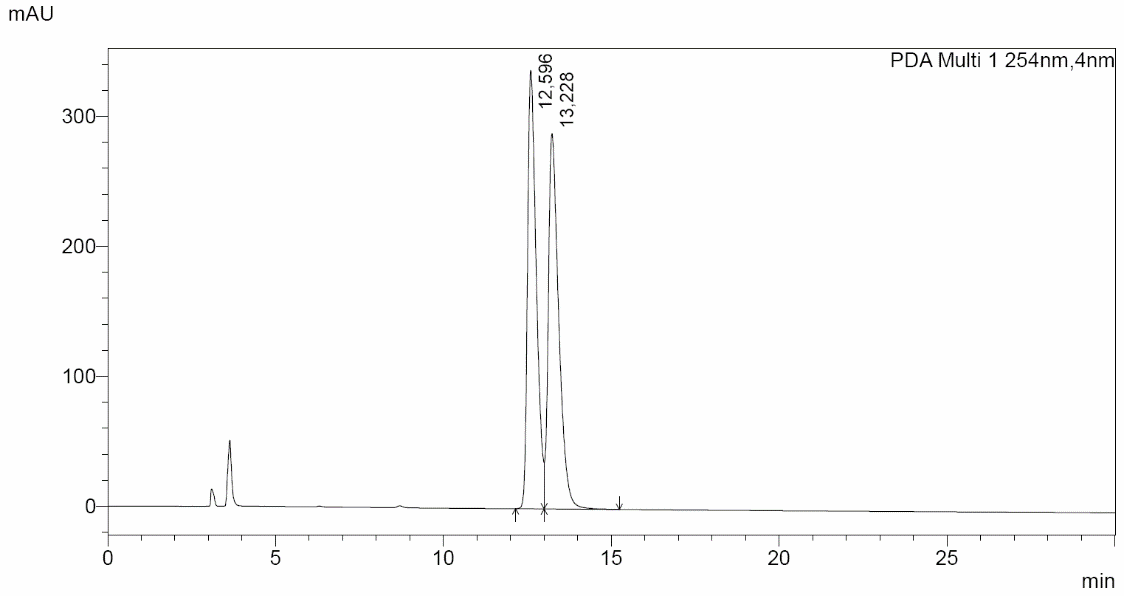

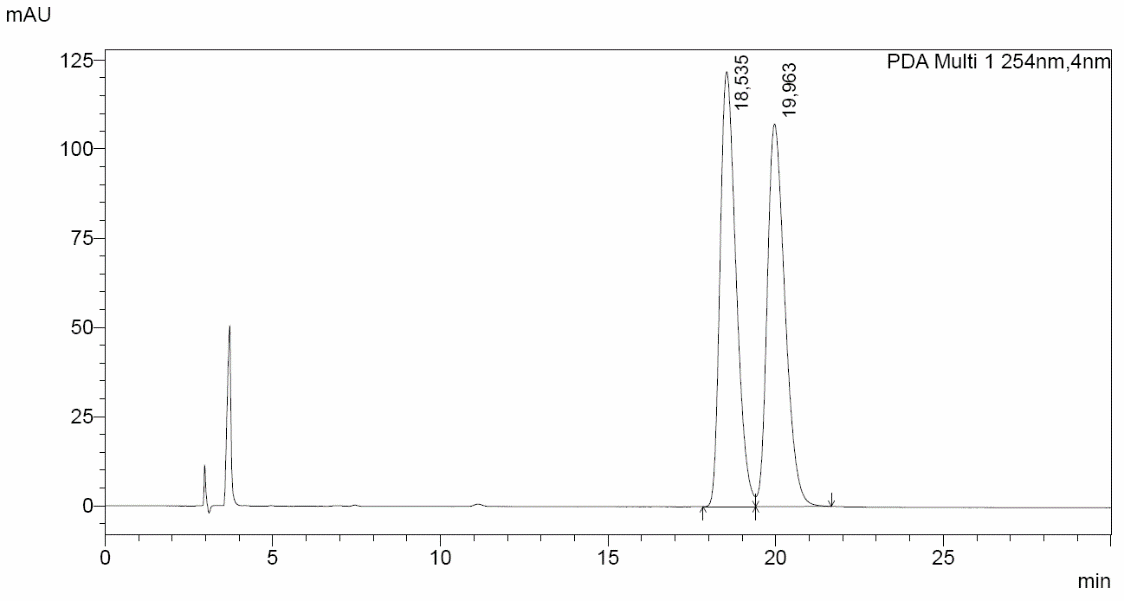

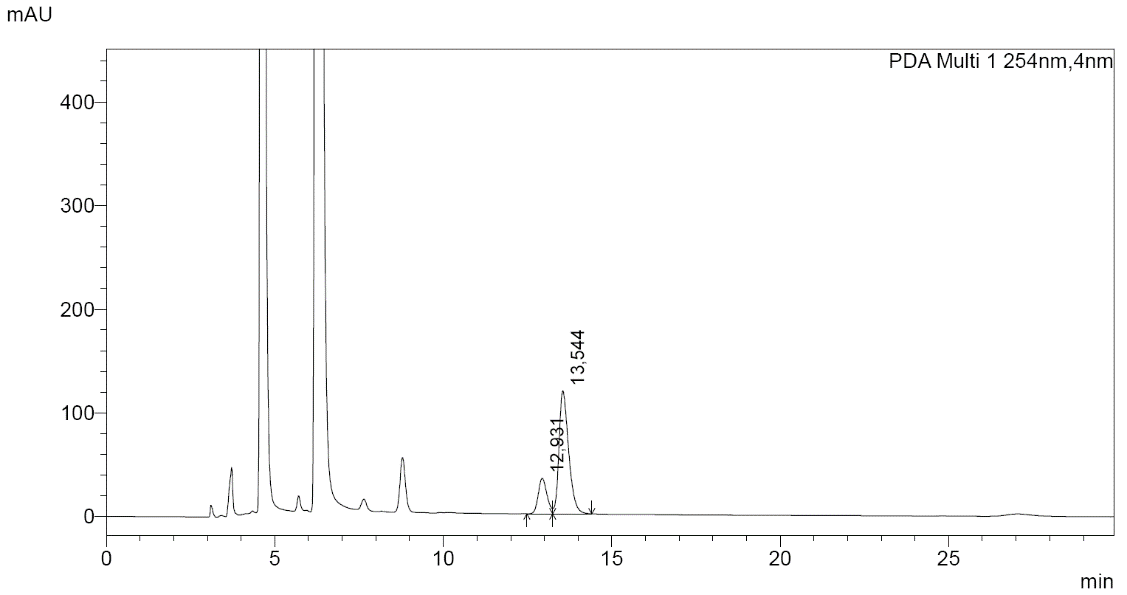

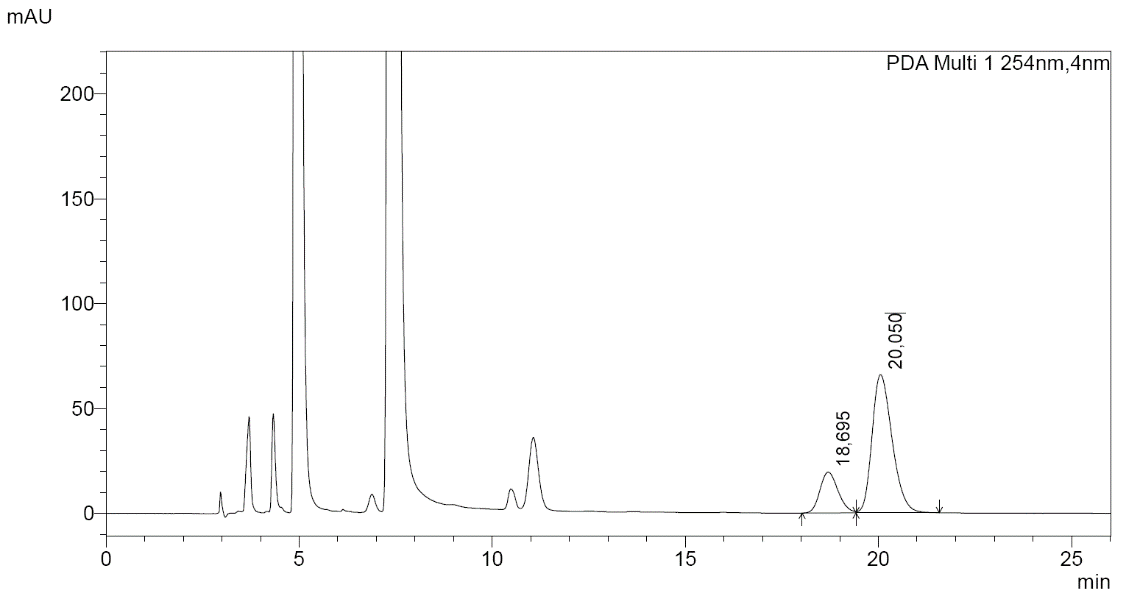


**a b**

**c d**

## Fig. S17. HPLC analysis for determination of methodol absolute configuration and enantiomeric excess.

**a.** and **b.** HPLC traces of freshly synthesized *rac*-methodol obtained on a Daicel Chiralpak IB column (**a**, retention times: (*S*)-methodol 12.6 min, (*R*)-methodol 13.2 min) and a Daicel Chiralcel OD-H column (**b**, retention times: (*S*)-methodol 18.5 min, (*R*)-methodol 20.0 min). **c**. and **d**. Representative traces from samples of the aldol reaction with RAD29 showing formation of the (*R*)-enantiomer with 60% ee on a Daicel Chiralpak IB column (**c**) and a Daicel Chiralcel OD-H column (**d**) obtained under the same conditions as in **a.** and **b**.


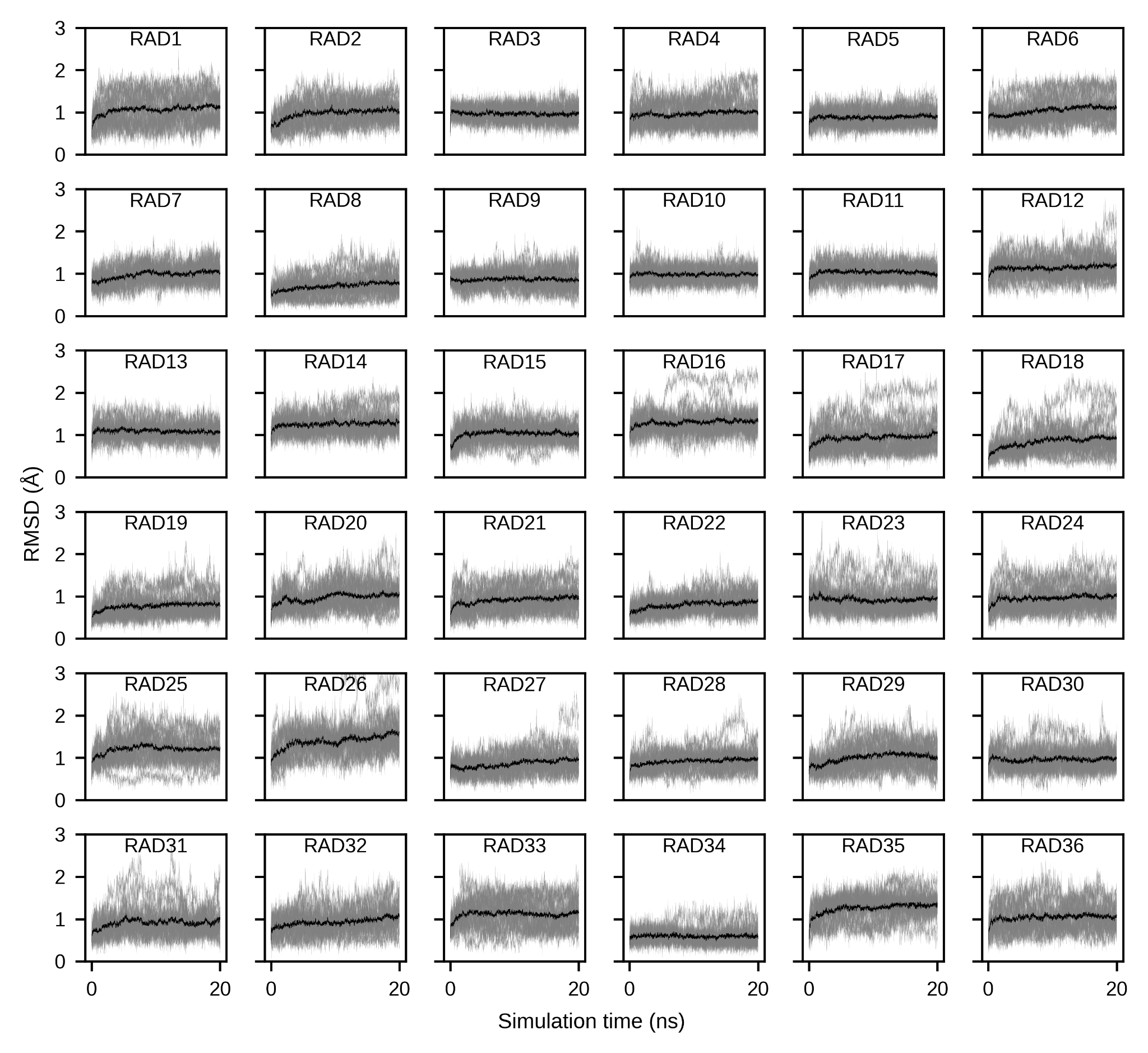


## Fig. S18. Backbone Cɑ RMSDs of the simulated RADs for all MD trajectories.

Grey lines represent RMSDs measured for each individual trajectory and black lines correspond to the average over all 20 trajectories.


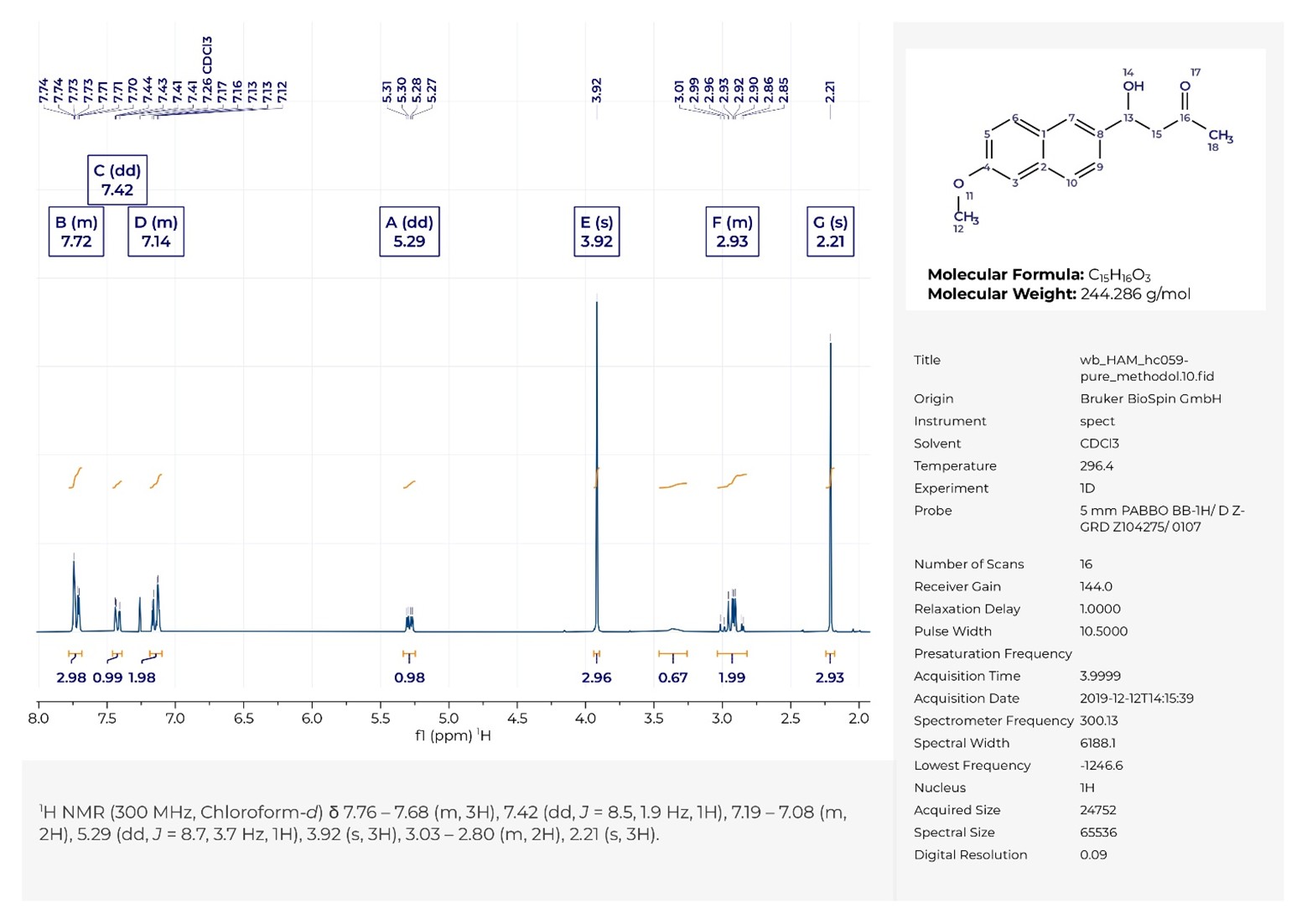


## Fig. S19. ^1^H NMR spectrum of *rac-*methodol 1.


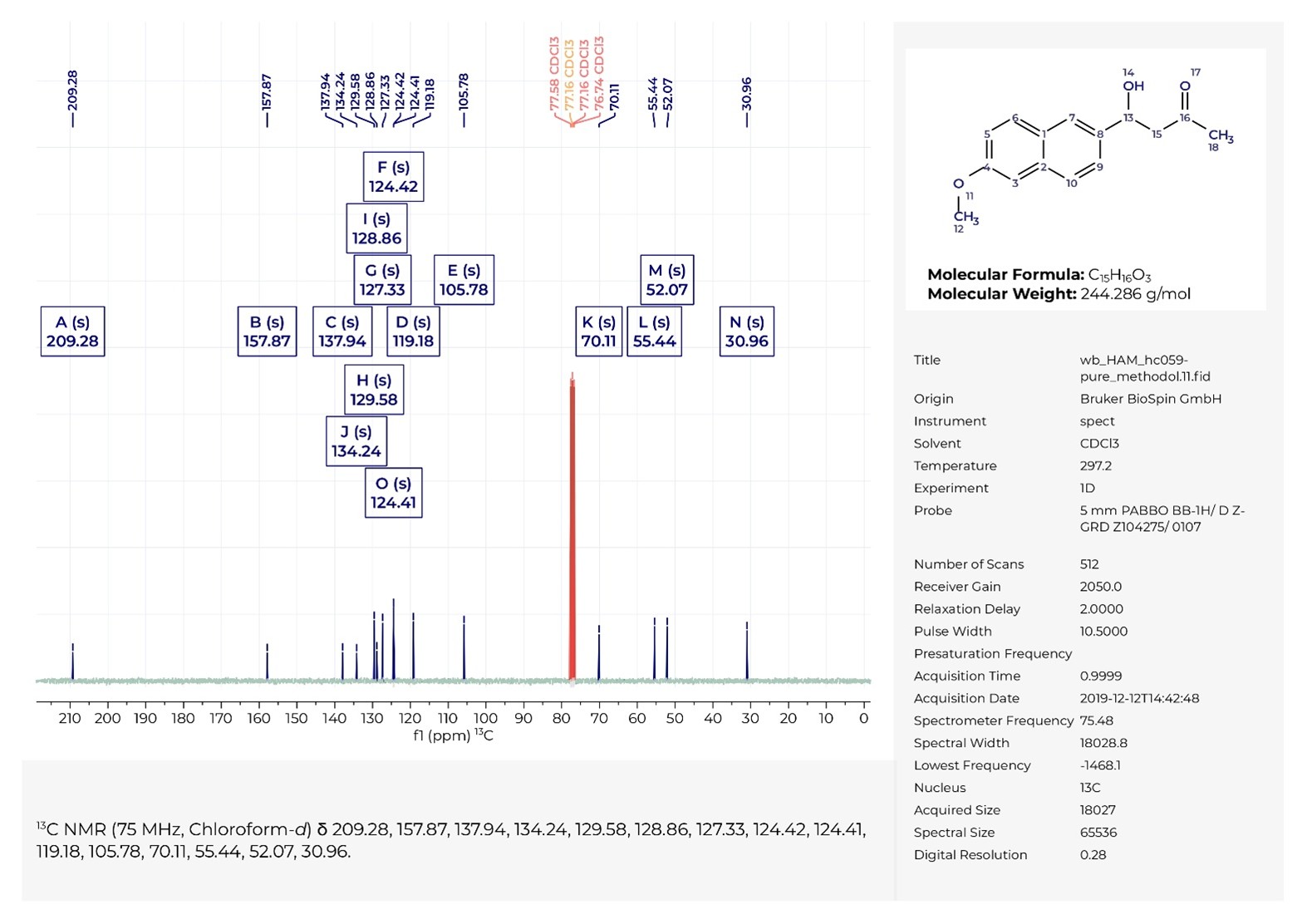


## Fig. S20. ^13^C NMR spectrum of *rac*-methodol 1.


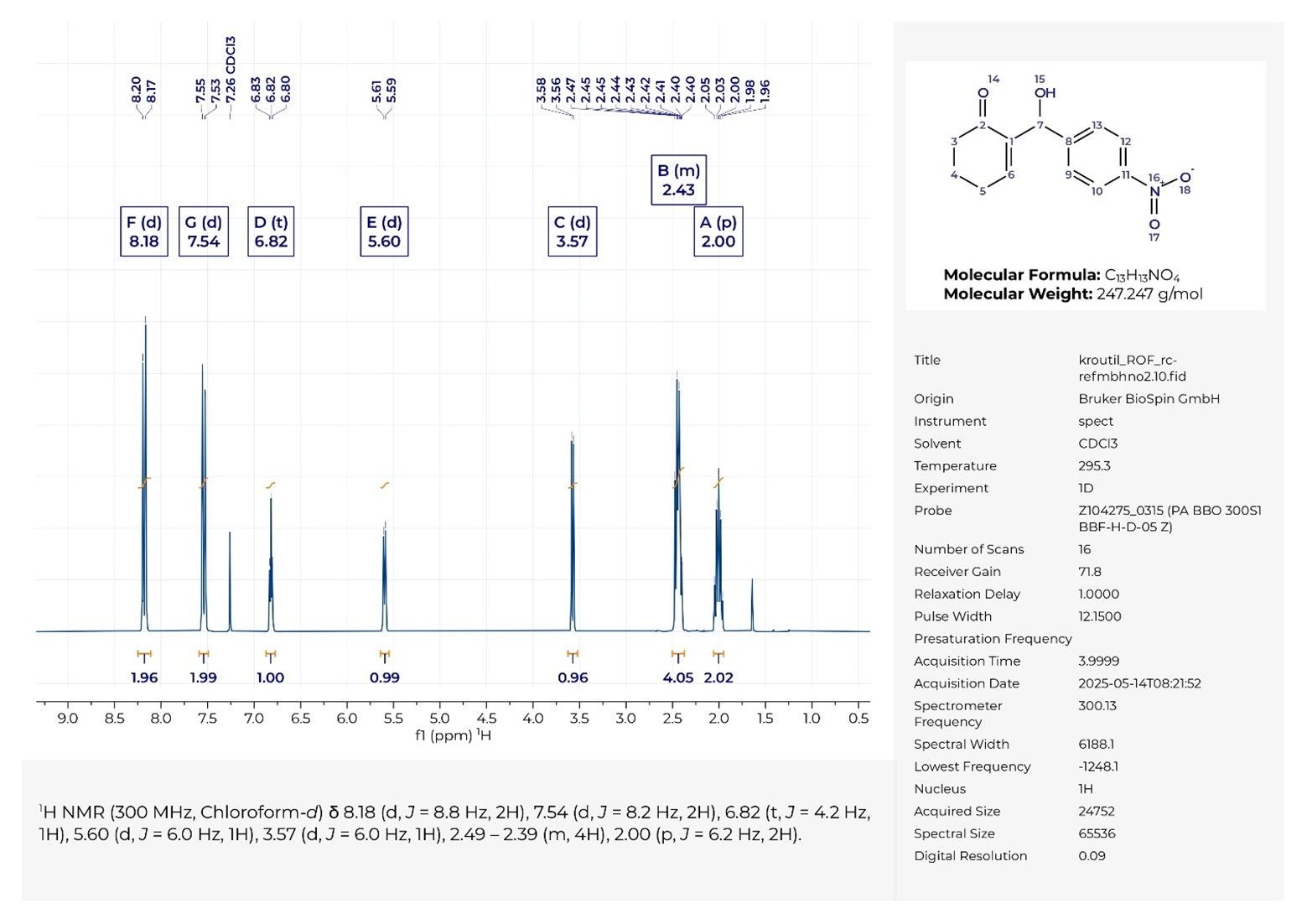


## Fig. S21. ^1^H NMR spectrum of 2-(hydroxy(4-nitrophenyl)methyl)cyclohex-2-en-1-one 5.


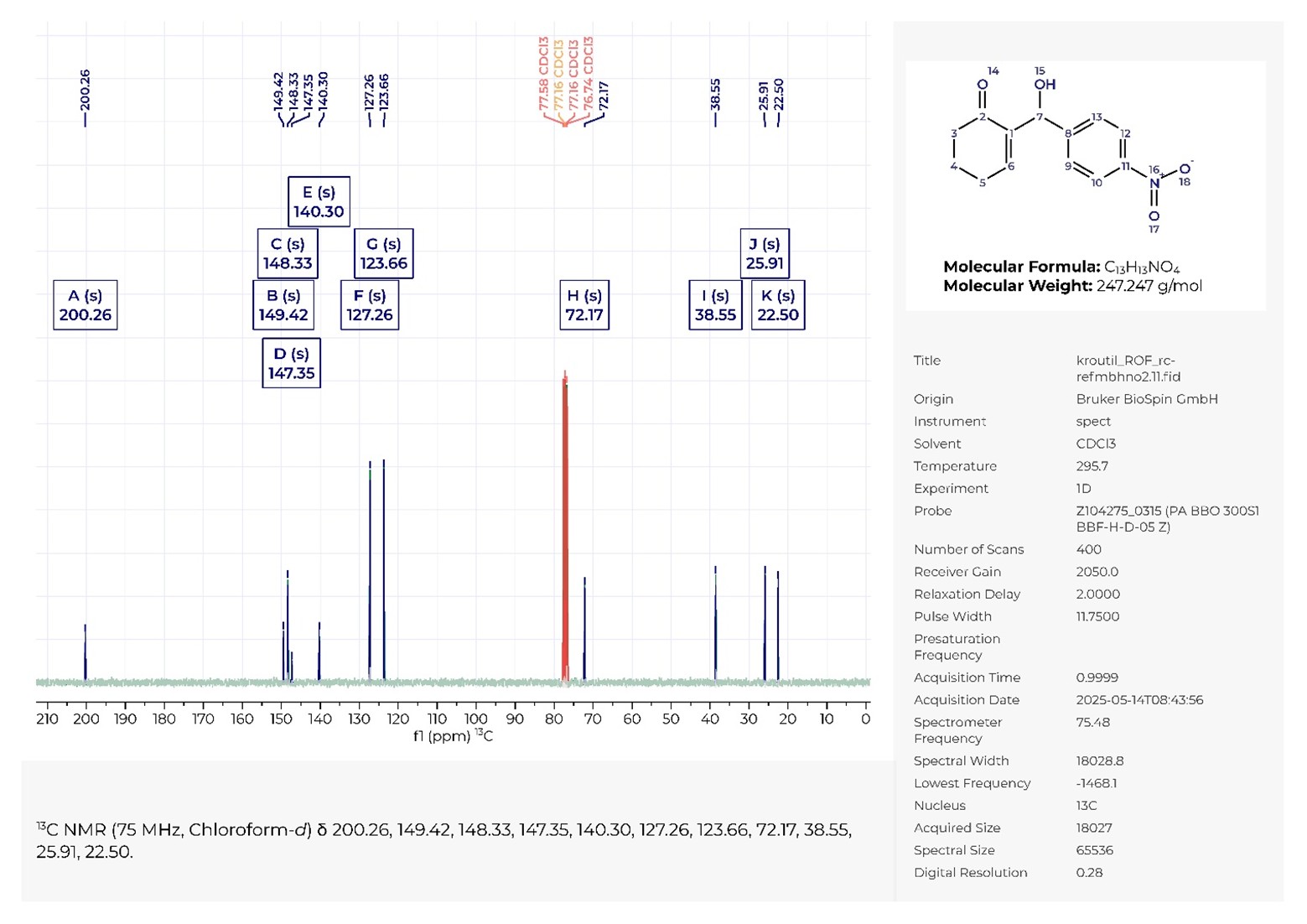


## Fig. S22. ^13^C NMR spectrum of 2-(hydroxy(4-nitrophenyl)methyl)cyclohex-2-en-1-one 5.


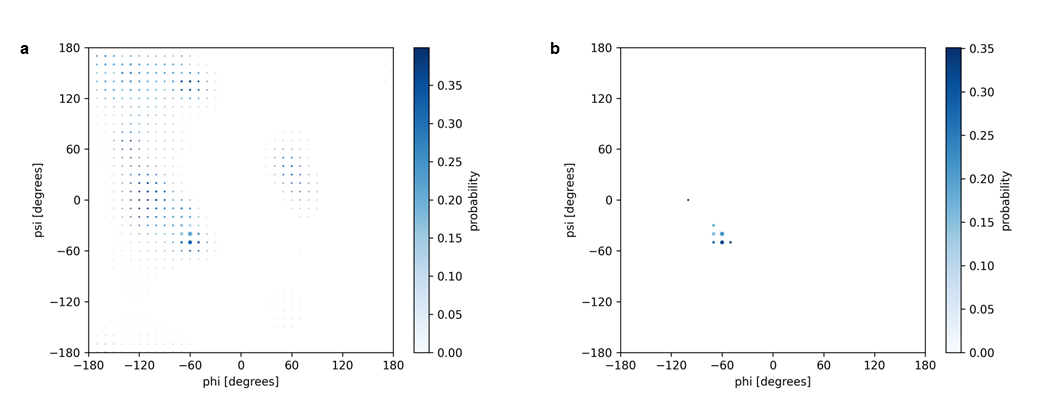


## Fig. S23. Selection of rotamers for the nucleophilic histidine residue in the MBH reaction.

**a,** Ramachandran plot of histidine. The color gradient indicates the probability of the most common rotamer at each phi/psi angle combination. Dot size corresponds to the occurrence of the phi/psi angle combination for this residue. Only rotamers on phi/psi angles in the helical region of the Ramachandran plot were considered for selection. All data was extracted from a previously published backbone-dependent rotamer library^32^. **b,** The top 15 rotamers according to probability and phi/psi angle occurrence were selected (multiple rotamers for a single phi/psi angle combination are possible).

# Supplementary Tables

## Table S1: Amino acid sequences of RAD constructs.

All genes were cloned using Golden Gate cloning into a vector featuring a N-terminal hexa-histidine tag and a TEV cleavage site with the sequence MGSSHHHHHHSSGENLYFQSG. Extinction coefficients were calculated using ProtParam. 6H5L_TOP10^75^, a recent de novo retro-aldolase developed in our lab, and IL-37 NOSMAD3, an unpublished modified version of interleukin 37 without enzymatic activity, were used as positive and negative controls, respectively.

| **RAD** | **sequence** | **extinction coefficient [M^-1^ cm^-1^]** |
| --- | --- | --- |
| 1 | MSEEEKRKKEEEEEYKKKLEEAREYLEKTMGKFTSLLLKAAGREDLIPTYAPLSSEYVKNVPDEKLLEIKEKNEKLGEKAKEKEEEFEEYKKLLLELASKDLSPEEAKLVGLGSLAFFLGVGKYEGMEKFYELWEEFKKVASPSVLDALREFFAGLYEKIAKSKTLEEFLEGVFEGYVEFMEFIFENYDELLEKVKELAK | 20400 |
| 2 | MSKEELEKLKKEEERKKEIEEARRYLERTMKRFTTLLLKAAGREDLIPELAPAASEYVKNIPDEELLEILKKNKELGEKAKKLEEEFEKLKETFLTLAGKDLSPEEARLVGLGSLAFFLGIGKAKGEEYFYELWEEFVKVASPAVLEALREFFRGLFEKINKAKTKEEFLKGVYDGYKEFMDFIFNNFEELVEKVLELSK | 14440 |
| 3 | MEKLKEILIELAKLAGDPSEESQKEYLGKAIAFMGALALAALDPSLQSPEALAELTEELLPLGEEGAKTLSEEEIKEITDFNLSMASYFGDMTPEELVAAVAAAPSALLALTIAVGGIKGVAVKDPSKVPEYVEAFLEGLGTWAAKHPEEWHAFFAAMGEEILADPSKLAENMAKVYKDLVDELKKLLPEAVKLYHEVKA | 18450 |
| 4 | MERLKELLIELAKLAGDPSEEKQREYLGKAIAFLGALALAALRPELQSPEALAELTEELLPLGEKGAETLSEEEFQFISDFNLSAADDFADATPEELVEAIVAAPSIELALAIAVLGIKGVAVKDASKVPEYVEAFLRGLGEWAKRHPEEVRAFFEGAGRRLVEDPSTLAQRMADLYKELVKRLKELRPQAKALLEEVKK | 9970 |
| 5 | MEKLKEILIELAKLAGDPSEESQREYLGKAIAFLGALALAALDPSRRSPEAIAQLTEELLPLGREGAKTLSEEEFKYITEFNLKAGEYFKDVTPEELVKAVVAAPSPELALTIAVSGIKGVAVEDKSKVPEYVDAFIEGLGEWAKKHPEEVAAFFEAMGRELLADPSTLAERMARVYRDLVKELEKLLPEAVALVEEVKK | 12950 |
| 6 | MEKVREILIELAKLAGDPSEEAQRKYLGKAIAFFAALALSALDPALRSPEALAALFEELLPVGEEGAEGLSEEEKEKIFRFNREVGLKFKDVTPEELVAAIVAADSPELALTIGLGAVKGHAVLDPSKVREYVEAFLRGLGEARARHPEAWDAFFRAAGRRLLADPSRLAERAAGLYADLVSRLESLLPEALALVEEVKK | 9970 |
| 7 | AEEEAKKIIEKGRKFMEEHGREYVEKVLEEVGKFLAKVAKDPVLLAGLTKLDPRFANLNLSREERLELYIWIAGFGNKVAAEALREEGLEKAAEIFEKASKKALKGVELAKEKGVEEAIKYALEANAENGGAALLALKESGKGVPLVERLLRKAKEEPEKAGEYFLGALVLGFSIIKGLAFDPDNEVLREAAERVRELLG | 11460 |
| 8 | LSPAEREELRRRLRLLAIFGAKGLLNRGEELEVTPEDEPYLERGRELAEKGIELYEEYPEVIDYLKPHLEELKRINEEESLEFLEVIEEAPEELQPLLRREMAFAYAALSTLREKAKELGEDEDLNYYGLAAVHQALMRVLEEVRREDPSLSEEEALEKAIERTRELLKNPKEIADYYVEATYRMLEEARALGEEIRGLH | 14900 |
| 9 | MEEALEASREAIELDPEGAKEVAELNREAGRIVEEAGSYEEVAKKVLEAAKEGKLSDETIIAAAKGLVYTPEGREVARKTAEEAEKLAKESEGEERKRLALLSFLLRLLVEIYEKSKDDEGYLVLAGVHWLAAKIAKKELEKNPEWYYDVEGIEEAFKKGLEEAAKAPREEIIKAGLDYFKEAKKIMEKGNKELKELLFK | 21430 |
| 10 | MEKALEAAREAIKENPEEAKEVAELNREAGKVVEEAGSYEEVAKKVLELAEKGKLSDDAIIAAAKGLTYTPEGVEVALKTAEEAKEKAKKSEGEGKERLTLLSFLLELQAELTLQSKDDEGYLTLVTVYWLAAKIAKEYLEKNPEASTDYEGIKEAFEKGLEEALKAPAEEIKKAGFDYFKNAKKILKKGNKELRELLFE | 15930 |
| 11 | MEKALEAARKAIEANPELAAKAAELNKKAGEIVEEAGSYEEVAKKILEAAKEGKLSYNAIIAAAKGLAYTPEGQKVALETAEKAEEEAKKSSGESKEKLILLSFLLRLQVKLKEESEDDEGYLTLATVYWLAAKIAKKKLEEDPSLAESLEGIEKAFKEGLEEAEKAPEEEIIKAGKDYFENAIGIMTKGNEELKKLLFS | 14440 |
| 12 | MEKALEASREAIKLNPELAKKVAELNEEAGRIVKEAGSYEEVAEKILEAAREGKLSRETIIAAAKGLVYTPEGQEVARATAERAEEAARESEGEGRERLTLLAFLLRLLVELYEASEDDEGYLVLAGVHWLAAQIAKKKLEENPEAALDIEAIEKAFEEGLEEAAKAPEEEIIKAGLDYFEEAQKIMEKGNKELKELLFS | 12950 |
| 13 | MEKALEAARKAIEEHPEEAKEVAELNKKAGEIVKEAGSYEEVAKKVLELAREGKLSDDAIIAAAKGLAYDEEGQEVALKTAEEARKAAEESSGKGKERLTLLSFLLRLQVRLTRESEDDEGYLTLATVYWLAAKIAKKKLEEDPSASTDLEGIEKAFEEGLEEAKKAPEEEILKAGFDYFEKAKEIMEKGNKELRELLFK | 12950 |
| 14 | FEKALKRGREFYEKDPEGFKKMMELNEELADLAYKLIEEGKIEKIVELAAEYYKKGDELTAALVLAALVKGIALHDLPEEYVEKAAEYAKKLIGEEWGEFIRGLVAFLRKLKEAFPDPEELHLAYFGYVTALFLAIERLKENPELYKDKPYFDRILLTAEEILNDESTLPELEKYAEFYLTEVMEKLEKIKKELEKILKE | 23380 |
| 15 | FEKALEYGRRFYEEDPEGFERMMKLNFELAKLAKELVEKGKIREVIELAAEYYKKGDELTAALVLAALVKGMALYDLPEEYVEEAAAYAEELIGPEWGEFIRGLVAFLRELKKAFPDPEELALAYFGFVTFLFLAIDELKKDPSLYADEPFFDRILKKAEELLNDESTLPKAREYARFYLTEVMEKLEEIRKKLEEILEK | 21890 |
| 16 | FEKALEWGRYFYAKDPEGFKKMMDLNEEMAKLGAELVKQGKIEEVVELAAEYYKKGDELTAALVLAVLVKAMALYDLPEEYVEKAAKLAEEKIGPEWGEFIRGLVAFLRELKAAFPDPEELALAYFGFVTALLLAIEELKKNPELYKDKPFFERILLTAEEILNDPSKLPELEKYAEFYLTEVMKKLEEIRKKMEEILKK | 25900 |
| 17 | MTPEEEARAAVDSFPEALRQRAWDLNVKSAEKLAKYGIEKVTELALKLLKEIFEKYVEGKITREDLPEVVKKILVLLSLVKATAIYSKEGLEKILELLKEIAKELRERGETLLAEAIDYLIEALEKLHKGDADGYLTLLTIALYLYFKHIVENGARDPELAAAVRPLVEGGYEAVARYYFEVFAPKLEEGTEEAVKLFEE | 20400 |
| 18 | MSDEELARAAVDSFPEAERQRAWDLNVKSAEKLAKFGIEKVTELALEKLKEIYKKFIEGKITKEDLPKLIEEILILLALIKATAIYSEEGLEKILEEYKKIAEELRKKGYTLLAEAIDYLIKALKALHAGDADGYLTLLTIALYLYFKHIVDNGKFDPELAEKVRPLVEGGYEAVARYYFEVFAPKLEEGTEKAVKLFEE | 21890 |
| 19 | MSDEEEAAAAVHSFPQELRDRAWNLNVESAEKLAKYGIEKVTERAVELLKELYENFVEGKITREDLPKVLEDLLVLLALVKATTIYSEEGLEKIIALLKEIAEKLRELGYTLLAEAIDYLIEALKKLHEGDADGYLTLLTIALYLYFKYIVENGHLDPELAALVKPLVEGGYEAVARYYFEVFAPKLEEGAEKAVKLFEE | 23380 |
| 20 | MTDLEKAREAVLSLPKELRERAWKLNVESAEELAKFGIEKVLEKALELTKELAELYFEGKITPEDLPEVAKKMLILLAYIKAATIKSKEGLEKIIKLYEEIAEELRKRGETLLAEAIEYLIKALEALHKGDAEGYLSLLTVALYKYYKWLVEKGDKFPELAALHKPLVEGGYEAVARYYFEVFAPAFEKGAKEAVESFKK | 27390 |
| 21 | MTDEERALAAVRSLPEELREYAWKLNVESAEKLEKFGIEKVTEIALKKLEELSELYFEGKITPEDLPEVFRKMLILLAYIKATAIKDEKGLEKILEKLREIAEELRKKGETLLAEALEILIEALKALHKGDADGYLSLLTIALHKYFQWVVSQGDKFPELAAAVQPLVDGGYEAVARYYFEVFAPKFEEGAKEAVEEFKK | 22920 |
| 22 | NLLEFWIKFLEGLVKSLSEEGREDYREGLGGLLRRAARGLEKLDPSLKEAIELVRRAAEKAGPNLTDEEILEINLEAGRAGKIVVEKGVEKVIDNGIKLIKENPLDLELLLEVTAALTVIAKGAYFDPSVVELLKKKAEELKKEGDDFTAFILELLAETGEKFAELAKEENGGEKYLEWLFTVVREEIEKKIEELKKKLE | 15470 |
| 23 | NLLEFWIEYLRELVDSLSEEGREVYKNSLGETLKLAAEGLKLADPSMKEAIELVEKAAEKAGPNLTDEEIGEINLKAGEAGEIVMKKGYKPVVDKAIELWKKNPLDKEILLKVTNAITVIAKGSYKYPEVVDYLREKAKELRKEGDDFLALILELLAETGEKWAELAKEKEGTKKYLEWLFSKVKGRIEEKIKELEKELK | 32430 |
| 24 | DLKKFYIEYLEGLVESLSEEGLKVYKEGLGELLTRAAEGLELLDPSLKEAIEKVREAAKKAGPNLTDEQIKEINLEAGEAGKIVIEKGYKPVVENGIKLIKENPLDLELLLKVTNALTVIAKGAVYNPEVVEYLKKKAEELEKEGDTFTAFILRLLAETGEKWAELAKEENGGKKYLEWLFTEVRKEIEERIEELKKELE | 21430 |
| 25 | NKELQEIADKIYNGLKSLSGKEYYEWVKNAPEKFEKNKKIFLEDPSKLDFDGFFTVITATLKGVLGKSKLEEELKKLAEIIEKTVSLLSKVNPFWLKLAEAGLLFEEIAKKLYKENPEIVEEYFESLGNTFEEALEVVEKLNTIPRGVLFILLAGMAIHASKFKELSKEEIEEVFEFNKKMAEIGLPFIEEALEALKKKE | 18450 |
| 26 | MEELVEKALELAPKKAKENRKPYFDFVMKYVEDPELLRRTLAEGARKFAEAVALRRGEEAERLLAEAAFAVIVGAKGYVVAGDKERLDELKAMLEEMAESVRTAQLLVAAFGIIEKLIDDPKKYIGAITAFLGTVEELEKEGPLSLEELKAIAEAIIENADKLVDVSDEEVAKMDAFNKRMGEIAIPLLEAALEALRASL | 5960 |
| 27 | MEEYEKILDEIVNGLRNMSGEEYYEMVLNAEKYYYPAVEKFLEDPSSLNGDEFFTVFTLSLKGVLAAGDLDTLLPKYAKIIEETIKEMSKKDPFWLDFAKAALKFEKVFQEEYEKNPEVVDEYFRANGVSPQEALDYALKFDDISRGVLLILLAGMAIQGLKLLSLSEEQREKVFAFNRSMKDIGLEFVDKALEALEKKK | 20400 |
| 28 | MEKLEELAKKILDFLESMSGQDYYEMVKNFEEYFEKAKKKFLEDPSKLNFGEFFTVITYTLKGVLGKADLEEEFPKLAEVIRETLEILSKKDPFWLDLAEAGLLFEEVAEKYYKEDPEIVEEYFSSLGVSFEEALEVALKFDKIPRGILLILLAGMAIHARKLKDLSEEERKEVFEFNKKMAEIGMKLVEEALKELEKKK | 15930 |
| 29 | MEELVEKALELAPEAAKENAQPYYDFVKKYIEDKELLKKEIEEGSKNFAKALELKEGEEAEELLKKSAFAVIVYAKGLALSGDEEKFEELLEKIKEMAESLETARLLYAALLIIKKLIKDPKKYFGAITAFLGVVERLEKERPLSLEELVAIAEAIIERAEELQAFTDEQVEEMRKFNEEAGKLAIEYFEKALEELKKKK | 10430 |
| 30 | MEELVKRALELAPRTAKERREPYFEFVSRYIDDPELFRREMEEGARLFEEALRLRTGPEAERLLREAAFAVIVGAKGFAVAGDEERFNRLVEALREMAEDVPTARLLVAAFEIIKRLIDDPEKYIGAVTAFLGVVEELEKERPLSLEELVAIAEAIIERADELVDVSKEEVERADAFNRRAGEVAVPYLDRALDAVEAER | 5960 |
| 31 | MMEKAEEAIKEAIENRDERVEPYVERAKKLEKEIKKYVEAGVEAIVQAIKDGDLLVLAMLLKGLFYHGFYGKEEEAIELLERLAEAVPTLEGRLMALLYAELLRYVKEKGISKEEFAPLYLTVILILLPTYKKLVEEGVLSEETSLEELREIIRLVIERTPEPSEKIKKATEEVEPINEKMGELLSFEEMKEVVEGVANS | 11920 |
| 32 | MLEKAKEAIKEAIENRDELVEPYVERAKKLAEEIKKYVEGGVEAIVEAIKNGDLEVLAMLLKGIFYHGFYGEREEAIELLEKLAKAVKNLEQRLMSLLYAELLRYMEEKGISWEEFAPQYLTLITILLPTYEKLKEAGVVTESTSLEELREIIKLVLENLPEPSELEKEATKEVEPINKKMGEYLSFEELKEVVEGVANG | 18910 |
| 33 | MMEEAERAIEEAIKEIDERAEKYVERAKWMIEEVEKYVEAGLQAVIDAILNGDMIVLAMFLKGIFFHGLKGKVEEAIELLRRLAEAVPYLEGRLIALMYALFLEYVTEKGLSLEESYPIYLSVILVLLPTFEKLVEKGVLSEETSLEELEELIRLVIEKTPEPSEKVKEYVKEVAPINEKMGELLSFEEGREVVEGVAAA | 17420 |
| 34 | MREAVEEALARARERRDELVGRYMAWAREFYNNPELQEEILEGAKEAIRDPSDPEKMKALGIALAIAIKGAVFSSEEEIERARRLLERLGKLSPEHAKLAEFMLALLDAMVTLRETLSREDFALAYLGLLMVVLLALSEGDVETATAALRLVAEGEYEPFLELAAPYREEAEEALRINEGLVPLGLRFLEELERVVEEVE | 12950 |
| 35 | MEEAALKAIEEAIKNREKIVKEYVERAKEMIEKLKEYVKKGKEAIVDAILNGDMDVLAMFVKGIFFHGLRGREDEAIELLERLAEAVKTLEGRLIFLTYAEMLRYIRERGISREEFWPLYLSLITVLLPTFERLVKEGVLSEETSLEELRDLIRLVWERTPEPSELERKATEEVAPINEEAAELISFEEGKEVVEGVASA | 18450 |
| 36 | MRERVEEALRRAKERREEIIGKYLEWAKTFANNPELQKEINERALKAIKDPSDEKDLKALGIALAIGMKGPIELGEEAVEELLGLLERLGKLSEKHAELADFLKALVQAYMTLKKTLSEEEYRVTYLGMIAVVLLALSEGDYDTAKAALELVVEGDYEPFLELAEPYAEEAKEAWEINKKLVEYGLKVLEKMKEAIKEVE | 22920 |
| 6H5L_TOP | GCEVYKKILYMAKKLVEKKKEVVKKILKMAKELVEKKKEVVYKALEAAEKGLDTKKIAKLLLEMLEHELELAEKIAKLLLEMLEEELKLAEKIAKLFLEEGAEEEVLKKWLYMAKKLVEKKKEVVKKILYMAKELVEKKKEVVKKALEAAEKGLDTKKIAKLLLEMLEHELELAEKIAKLLLEMLEEELKLAEKTAKLKLESGASEEIQKKYLKMAKKLVEKKKEVVKKILYMAKELVEKKKEVVKKALEAAEYGLDTKKIAKLLLEMLEHELELAEKIAKLLLEMLEEELKLAEKYAKLYEEQCK | 20525 |
| IL37-NOSMAD3 | GVHTSPKVKNLNPKKFIIEDLNNKVLVLDSGNLIAVPKKPYIRPEIFFALASSLSSASAEKGSPILLGVSKGEFCLYCDKDKGQSHPSLQLKKEKLMKLAAQKESARRPFIFYRKKEGEYNLLESAAHPGWYICTSINCNEPVGVTDKDEKRKHKKFKFTPVCKAEMSPSEVSD | 12950 |

## Table S2. Mass spectrometry results of purified enzymes.

The expected molecular weight includes the serine-glycine stub that remains after cleavage of the histidine tag. For RAD14 and 15 this cleavage was incomplete, resulting in a higher detected molecular weight. The highest intensity peaks for each sample are displayed.

| **RAD** | **expected MW [Da]** | **detected MW [Da]** | **difference expected – detected [Da]** | **Intensity** |
| --- | --- | --- | --- | --- |
| **1** | 23569.65 | 23569.1 | -0.55 | 898358 |
| **2** | 23536.89 | 23536.4 | -0.49 | 1552472 |
| **3** | 21670.68 | 21670.3 | -0.38 | 869407 |
| **4** | 22140.16 | 22139.7 | -0.46 | 880003 |
| **5** | 22113.19 | 22112.8 | -0.39 | 479635 |
| **6** | 21888.87 | 21888.3 | -0.57 | 1287525 |
| **7** | 22219.38 | 22218.8 | -0.58 | 1140131 |
| **8** | 23571.27 | 23570.7 | -0.57 | 1373084 |
| **9** | 22658.54 | 22658.3 | -0.24 | 1371438 |
| **10** | 22359.14 | 22358.7 | -0.44 | 1251987 |
| **11** | 22022.86 | 22022.4 | -0.46 | 1514071 |
| **12** | 22286.9 | 22286.4 | -0.5 | 1379381 |
| **13** | 22435.1 | 22434.7 | -0.4 | 1472734 |
| **14** | 23538.93 | 25618.9 | 2079.97 | 609901 |
| **15** | 23491.77 | 25571.7 | 2079.93 | 582547 |
| **16** | 23285.91 | 23285.5 | -0.41 | 432873 |
| **17** | 22817.1 | 22816.8 | -0.3 | 1184266 |
| **20** | 22932.46 | 22931.9 | -0.56 | 1579678 |
| **21** | n.d. | n.d. | n.d. | n.d. |
| **22** | 22664.84 | 22664.5 | -0.34 | 1445679 |
| **23** | 22926.24 | 22926.2 | -0.04 | 1074630 |
| **24** | 22777.9 | 22777.7 | -0.2 | 1691445 |
| **25** | 23115.53 | 23115.1 | -0.43 | 348040 |
| **26** | 22236.5 | 22236.2 | -0.3 | 1418615 |
| **27** | 23162.25 | 23161.8 | -0.45 | 1202863 |
| **28** | 23505.96 | 23505.5 | -0.46 | 1027103 |
| **29** | 23068.37 | 23067.8 | -0.57 | 1292346 |
| **30** | 22920.77 | 22920.6 | -0.17 | 2171495 |
| **31** | 23043.51 | 23043.1 | -0.41 | 721705 |
| **32** | 23038.34 | 23037.9 | -0.44 | 1392019 |
| **33** | 22899.36 | 22898.9 | -0.46 | 369553 |
| **34** | 22729.77 | 22729.7 | -0.07 | 1379768 |
| **35** | 23187.47 | 23187 | -0.47 | 1061567 |
| **36** | 22885.14 | 22885 | -0.14 | 1450476 |

## Table S3. SAXS parameters.

%MW and %Rg denotes deviation from expected molecular weight and Rg calculated from the design models.

| **RAD** | **FoXS chi²** | **FoXS c1** | **FoXS c2** | **FoXS default chi²** | **BI MW [Da]** | **%MW** | **Size& Shape MW [Da]** | **%MW** | **Rg Guinier [A]** | **%Rg** | **I0 Guinier** | **Rg p(r) [A]** | **%Rg** | **I0 p(r)** | **Rmax (p(r)) [A]** | **Porod volume [A³]** | **Porod Volume %** | **model Rg [A]** | **model**  **Porod**  **Volume [A³]** |
| --- | --- | --- | --- | --- | --- | --- | --- | --- | --- | --- | --- | --- | --- | --- | --- | --- | --- | --- | --- |
| **1** | 2.3 | 1.04 | -0.22 | 56.5 | 18675 | 79% | 22769 | 97% | 20.4 | 100% | 82.23 | 20.6 | 101% | 82.36 | 70 | 40995 | 140% | 20.44 | 29300 |
| **2** | 1.2 | 1.04 | 0.05 | 35.6 | 18050 | 77% | 22625 | 96% | 20.2 | 99% | 72.11 | 20.2 | 99% | 71.87 | 60.7 | 39406 | 148% | 20.44 | 26710 |
| **3** | 5.3 | 1.02 | 4.00 | 167.4 | 15475 | 71% | 20906 | 96% | 18.7 | 118% | 74.82 | 18.3 | 116% | 74.09 | 53.4 | 30020 | 119% | 15.82 | 25280 |
| **4** | 2.3 | 1.03 | 2.92 | 78.4 | 16125 | 73% | 26379 | 119% | 18.8 | 117% | 54.44 | 18.5 | 115% | 54.15 | 56.9 | 29298 | 109% | 16.06 | 26770 |
| **5** | 39.5 | 1.03 | 4.00 | 311.7 | 16125 | 73% | 23062 | 104% | 20.5 | 127% | 90.13 | 20.6 | 128% | 90.1 | 70.6 | 40165 | 161% | 16.09 | 24930 |
| **6** | 4.4 | 1.01 | 4.00 | 57.8 | 14825 | 68% | 19010 | 87% | 19.2 | 121% | 49.84 | 19.2 | 121% | 49.7 | 64.9 | 30541 | 126% | 15.9 | 24240 |
| **7** | 4.4 | 1.01 | 4.00 | 57.8 | 14150 | 64% | 19163 | 86% | 19.5 | 118% | 46.24 | 19.6 | 119% | 46.14 | 64.6 | 31931 | 140% | 16.49 | 22800 |
| **8** | 8.7 | 1.01 | 4.00 | 62.4 | 20550 | 87% | 23865 | 101% | 18.5 | 110% | 65.05 | 18.4 | 109% | 65.09 | 53.7 | 36386 | 118% | 16.85 | 30830 |
| **9** | 1.6 | 1.02 | 1.94 | 58.6 | 15475 | 68% | 21417 | 95% | 18.5 | 111% | 68.58 | 18.1 | 109% | 70.18 | 51.7 | 31146 | 126% | 16.68 | 24800 |
| **10** | 1.2 | 0.99 | 3.91 | 64.2 | 15475 | 69% | 19492 | 87% | 18.1 | 108% | 76.92 | 18.3 | 109% | 68.44 | 56.4 | 30491 | 110% | 16.73 | 27690 |
| **11** | 1.1 | 1.01 | 1.94 | 46.7 | 14825 | 67% | 17008 | 77% | 18.1 | 109% | 76.92 | 17.9 | 108% | 76.77 | 51.5 | 30660 | 108% | 16.64 | 28340 |
| **12** | 1.4 | 1.01 | 3.61 | 56.3 | 12750 | 57% | 20623 | 93% | 18.6 | 111% | 72.12 | 18.4 | 110% | 72.0700 | 55.8 | 30681 | 134% | 16.71 | 22940 |
| **13** | 1.1 | 0.99 | 3.87 | 43.0 | 13450 | 60% | 18462 | 82% | 18.3 | 110% | 62.33 | 18.1 | 109% | 62.1900 | 55.2 | 29068 | 106% | 16.68 | 27330 |
| **14** | 8.1 | 1.01 | 4.00 | 116.0 | 16125 | 69% | 20722 | 88% | 19.6 | 119% | 67.23 | 19.1 | 116% | 66.0800 | 57.8 | 33931 | 115% | 16.46 | 29380 |
| **15** | 6.0 | 1.03 | 4.00 | 109.9 | 13450 | 57% | 16186 | 69% | 19.2 | 118% | 57.48 | 18.6 | 115% | 56.8000 | 50.3 | 34180 | 118% | 16.24 | 29010 |
| **16** | 2.4 | 1.02 | 4.00 | 62.6 | 19925 | 86% | 25396 | 109% | 19.5 | 119% | 57.05 | 18.9 | 115% | 56.3800 | 53.1 | 31014 | 107% | 16.38 | 29080 |
| **17** | 2.6 | 1.01 | 4.00 | 57.2 | 23675 | 104% | 24839 | 109% | 19.8 | 120% | 62.11 | 19.3 | 117% | 61.2700 | 56.6 | 35300 | 110% | 16.5 | 31970 |
| **18** | 1.7 | 1.03 | 1.15 | 32.0 | 17425 | 76% | 19323 | 84% | 18.6 | 113% | 47.37 | 18.3 | 111% | 47.2400 | 52 | 30492 | 97% | 16.52 | 31460 |
| **19** | 1.6 | 1.02 | 2.68 | 36.3 | 17425 | 76% | 24283 | 107% | 19.1 | 116% | 42.96 | 18.6 | 113% | 42.6500 | 55.4 | 32238 | 104% | 16.45 | 30980 |
| **20** | 1.2 | 1.04 | 0.06 | 22.3 | 18050 | 79% | 20954 | 91% | 18.4 | 111% | 44.26 | 18.1 | 110% | 44.1100 | 55.2 | 27037 | 85% | 16.51 | 31740 |
| **21** | n.d | n.d | n.d | n.d. | n.d. | n.d. | n.d. | n.d. | n.d. | n.d. | n.d. | n.d. | n.d. | n.d. | n.d. | n.d. | n.d. | n.d. | n.d. |
| **22** | 1.2 | 1.04 | 0.94 | 55.2 | 17425 | 77% | 21821 | 96% | 18.5 | 112% | 62.2 | 18.2 | 110% | 62.04 | 54.9 | 29807 | 102% | 16.54 | 29200 |
| **23** | 2.3 | 1.02 | 4.00 | 26.1 | 12025 | 52% | 20084 | 88% | 20 | 121% | 27.97 | 19.5 | 118% | 27.5500 | 58.1 | 26666 | 92% | 16.58 | 29080 |
| **24** | 1.9 | 1.02 | 3.03 | 83.4 | 15475 | 68% | 20592 | 90% | 18.9 | 114% | 58.39 | 18.7 | 112% | 58.2100 | 64.8 | 32596 | 117% | 16.63 | 27930 |
| **25** | 4.9 | 1.00 | 4.00 | 77.9 | 16775 | 73% | 19283 | 83% | 19.5 | 118% | 54.98 | 19.4 | 117% | 54.6800 | 67.9 | 29514 | 100% | 16.55 | 29600 |
| **26** | 1.5 | 1.04 | 0.08 | 43.9 | 14150 | 64% | 20927 | 94% | 18.3 | 109% | 65.11 | 18.1 | 108% | 65.0700 | 52.1 | 29963 | 106% | 16.75 | 28270 |
| **27** | 9.9 | 1.01 | 4.00 | 318.4 | 15475 | 67% | 20809 | 90% | 19 | 115% | 128.59 | 18.8 | 114% | 128.0000 | 60.4 | 36547 | 128% | 16.47 | 28650 |
| **28** | 9.7 | 1.01 | 4.00 | 292.3 | 22425 | 95% | 25748 | 110% | 19.5 | 119% | 136.86 | 19.3 | 118% | 135.9 | 66.3 | 40775 | 149% | 16.38 | 27360 |
| **29** | 1.4 | 1.01 | 1.88 | 30.0 | 16125 | 70% | 22828 | 99% | 18.9 | 111% | 68.73 | 18.6 | 109% | 68.5000 | 60.9 | 31562 | 101% | 16.99 | 31330 |
| **30** | 1.7 | 1.01 | 1.13 | 17.0 | 14150 | 62% | 17680 | 77% | 18 | 107% | 73.84 | 17.8 | 106% | 73.7600 | 58.6 | 26777 | 87% | 16.81 | 30940 |
| **31** | 1.5 | 0.99 | 4.00 | 39.0 | 18675 | 81% | 18878 | 82% | 18.4 | 112% | 65.45 | 18.1 | 110% | 65.2200 | 57 | 28780 | 99% | 16.48 | 29020 |
| **32** | 1.6 | 0.99 | 3.71 | 56.4 | 17425 | 76% | 18043 | 78% | 18.1 | 111% | 91.11 | 17.9 | 110% | 91.0600 | 48.7 | 29801 | 102% | 16.3 | 29130 |
| **33** | 1.8 | 1.00 | 3.18 | 88.3 | 17425 | 76% | 18264 | 80% | 18.3 | 112% | 77.34 | 18 | 111% | 77.1900 | 52.2 | 32135 | 110% | 16.27 | 29200 |
| **34** | 1.3 | 1.03 | 0.64 | 80.9 | 15475 | 68% | 17680 | 78% | 18.2 | 112% | 79.02 | 17.7 | 108% | 78.5200 | 70.1 | 27927 | 94% | 16.32 | 29630 |
| **35** | 2.5 | 1.02 | 1.26 | 85.4 | 15475 | 67% | 17737 | 76% | 17.6 | 109% | 105.12 | 17.3 | 108% | 104.8000 | 57.3 | 28979 | 105% | 16.08 | 27540 |
| **36** | 1.2 | 1.02 | 2.80 | 40.8 | 12025 | 53% | 19237 | 84% | 18.4 | 112% | 55.63 | 18.2 | 110% | 55.5600 | 51.1 | 30402 | 99% | 16.49 | 30710 |

## Table S4. RAD kinetics, denaturation midpoints, yield, and pKa1

Michaelis Menten parameters for the retro-aldol reaction with *rac*-methodol, chemical denaturation midpoints, expression levels and pKa1 of retro-aldolases. ± indicates 95% confidence interval of the fit. Measurements were performed in triplicate. Biological replicates are marked as repl. 2.

| **RAD** | ***k*_cat_ [s^-1^]** | ***K*_M_ [µM]** | **denaturation midpoint  [M GdnHCl]** | **yield  [mg/L culture]** | **p*K*a1** |
| --- | --- | --- | --- | --- | --- |
| **1** | 5.7E-05 ± 0.3E-05 | 710 ± 80 | n.d. | 16.3 | n.d. |
| **2** | 1.6E-05 ± 0.1E-05 | 190 ± 20 | 4.7 ± 0.1 | 16 | n.d. |
| **3** | n.d. | n.d. | 5.3 ± 0.1 | 2 | n.d. |
| **4** | 4.0E-05 ± 0.4E-05 | 1000 ± 200 | 4.4 ± 0.1 | 3.7 | n.d. |
| **5** | 2.6E-05 ± 1.6E-05 | 1900 ± 1600 | 4.5 ± 0.3 | 7.9 | n.d. |
| **6** | 2.80E-05 ± 0.4E-05 | 600 ± 150 | 4.50 ± 0.01 | 17.4 | n.d. |
| **7** | 3.6E-05 ± 0.5E-05 | 870 ± 200 | 2.5 ± 0.2 | 16 | n.d. |
| **8** | 3.3E-04 ± 0.1E-04 | 520 ± 50 | 3.3 ± 0.2 | 21 | n.d. |
| **9** | 3.1E-04 ± 0.1E-04 | 340 ± 30 | 3.5 ± 0.2 | 12 | n.d. |
| **10** | 3.6E-05 ± 0.2E-05 | 390 ± 50 | 2.7 ± 0.1 | 5.2 | n.d. |
| **11** | 6.8E-05 ± 1.1E-05 | 330 ± 130 | 3.1 ± 0.1 | 9 | n.d. |
| **12** | 1.2E-04 ± 0.1E-04 | 150 ± 50 | 3.1 ± 0.2 | 17.5 | n.d. |
| **13** | 3.8E-05 ± 0.5E-05 | 320 ± 110 | 2.6 ± 0.2 | 9 | 9.0 ± 0.2 |
| **14** | 5.6E-06 ± 1.4E-06 | 440± 240 | 6.1 ± 0.1 | 1 | n.d. |
| **15** | 7.9E-06 ± 1.2E-06 | 640 ± 190 | 6.5 ± 0.1 | 6.5 | n.d. |
| **16** | n.d. | n.d. | n.d. | 1.5 | n.d. |
| **17** | n.d. | n.d. | n.d. | 28 | 7.4 ± 0.1 |
| **18** | n.d. | n.d. | n.d. | 3.4 | n.d. |
| **19** | 3.6E-06 ± 0.3E-06 | 410 ± 90 | n.d. | 2.4 | n.d. |
| **20** | 2.6E-05 ± 0.2E-05 | 990 ± 110 | n.d. | 20 | n.d. |
| **21** | n.d. | n.d. | n.d. | n.d. | n.d. |
| **22** | 3.6E-04 ± 0.5E-04 | 590 ± 170 | 4.7 ± 0.1 | 3.4 | n.d. |
| **23** | 6.5E-05 ± 0.5E-05 | 710 ± 110 | n.d. | 4 | n.d. |
| **24** | 3.7E-04 ± 0.5E-04 | 210 ± 80 | 3.2 ± 0.1 | 14 | n.d. |
| **25** | 4.5E-05 ± 0.5E-05 | 280 ± 70 | 4.2 ± 0.2 | 32 | n.d. |
| **26** | 1.1E-03 ± 0.2E-03 | 68 ± 35 | 3.4 ± 0.3 | 40 | n.d. |
| **27** | 2.4E-04 ± 0.1E-04 | 660 ± 70 | 3.2 ± 0.2 | 28 | n.d. |
| **28** | n.d. | n.d. | 4.5 ± 0.2 | 14 | n.d. |
| **29** | 3.1E-02 ± 0.2E-02 | 110 ± 20 | 5.1 ± 0.1 | 18 | 7.0 ± 0.2 |
| **30** | 1.5E-02 ± 0.2E-02 | 210 ± 90 | n.d. | 32 | n.d. |
| **31** | 1.8E-03 ± 0.2E-03 | 670 ± 160 | n.d. | 11 | n.d. |
| **32** | 1.1E-02 ± 0.1E0-2 | 1400 ± 120 | n.d. | 22 | 8.0 ± 0.3 |
| **33** | 2.2E-04 ± 0.2E-04 | 940 ± 50 | n.d. | 16 | n.d. |
| **34** | 2.3E-03 ± 0.4E-03 | 190 ± 80 | n.d. | 22 | n.d. |
| **35** | 3.7E-02 ± 0.1E-02 | 350 ± 30 | n.d. | 16 | 7.6 ± 0.1 |
| **36** | 6.2E-05 ± 0.2E-05 | 540 ± 40 | n.d. | 27 | 8.6 ± 0.3 |
| **29 N178A** | 9.6E-04 ± 0.4E-04 | 120 ± 10 | n.d. | n.d. | n.d. |
| **29 Y120F** | 7.8E-03 ±1.6E-03 | 100 ± 40 | n.d. | n.d. | n.d. |
| **29 Y23F** | 1.2E-02 ± 0.1E-02 | 65 ± 9 | n.d. | n.d. | n.d. |
| **32 Y120F** | 2.3E-04 ± 0.5E-4 | 960 ± 340 | n.d. | n.d. | n.d. |
| **32 Y99F** | 7.8E-04 ± 0.9E-04 | 190 ± 60 | n.d. | n.d. | n.d. |
| **32 N178A** | 3.6E-03 ± 0.5E-03 | 330 ± 120 | n.d. | n.d. | n.d. |
| **35 N178A** | 1.6E-03 ± 0.2E-03 | 350 ± 90 | n.d. | n.d. | n.d. |
| **35 Y120F** | 8.4E-04 ± 0.5E-04 | 620 ± 80 | n.d. | n.d. | n.d. |
| **35 Y23F** | 1.0E-02 ± 0.1E-02 | 870 ± 120 | n.d. | n.d. | n.d. |
| **13 repl. 2** | 5.2E-05 ± 0.2E05 | 190 ± 20 | n.d. | n.d. | n.d. |
| **17 repl. 2** | 6.5E-05 ± 0.7E-05 | 220 ± 60 | n.d. | n.d. | n.d. |
| **29 repl. 2** | 2.2E-02 ± 0.1E-02 | 110 ± 10 | n.d. | n.d. | n.d. |
| **32 repl. 2** | 3.8E-02 ± 0.5E-02 | 1300 ± 300 | n.d. | n.d. | n.d. |
| **35 repl. 2** | 3.3E-02 ± 0.4E-02 | 580 ± 150 | n.d. | n.d. | n.d. |
| **36 repl. 2** | 5.6E-5 ± 0.1E-05 | 210 ± 10 | n.d. | n.d. | n.d. |

## Table S5. Comparison of designed and evolved Retro-aldol enzymes.

|  | **substrate** | **pH** | ***k*_cat_ (s^-1^)** | ***k*_cat_ /K_M_ (M^-1^ s^-1^)** | ***k*_cat_/*k*_uncat_** | **Ref.** |
| --- | --- | --- | --- | --- | --- | --- |
| **Computationally designed retro aldol enzymes (this work)** | | | | | | |
| RAD1 | *rac*-methodol | 7.4 | 5.70E-05 | 8.02E-02 | 8.77E+03 | this work |
| RAD2 | *rac*-methodol | 7.4 | 1.60E-05 | 8.21E-02 | 2.46E+03 | this work |
| RAD3 | *rac*-methodol | 7.4 | n.d. | n.d. | n.d. | this work |
| RAD4 | *rac*-methodol | 7.4 | 4.03E-05 | 4.00E-02 | 6.20E+03 | this work |
| RAD5 | *rac*-methodol | 7.4 | 2.59E-05 | 1.37E-02 | 3.98E+03 | this work |
| RAD6 | *rac*-methodol | 7.4 | 2.80E-05 | 4.68E-02 | 4.30E+03 | this work |
| RAD7 | *rac*-methodol | 7.4 | 3.56E-05 | 4.09E-02 | 5.48E+03 | this work |
| RAD8 | *rac*-methodol | 7.4 | 3.29E-04 | 6.34E-01 | 5.06E+04 | this work |
| RAD9 | *rac*-methodol | 7.4 | 3.06E-04 | 8.97E-01 | 4.71E+04 | this work |
| RAD10 | *rac*-methodol | 7.4 | 3.59E-05 | 9.18E-02 | 5.52E+03 | this work |
| RAD11 | *rac*-methodol | 7.4 | 6.76E-05 | 2.05E-01 | 1.04E+04 | this work |
| RAD12 | *rac*-methodol | 7.4 | 1.23E-04 | 8.26E-01 | 1.89E+04 | this work |
| RAD13 | *rac*-methodol | 7.4 | 3.75E-05 | 1.17E-01 | 5.77E+03 | this work |
| RAD14 | *rac*-methodol | 7.4 | 5.60E-06 | 1.26E-02 | 8.62E+02 | this work |
| RAD15 | *rac*-methodol | 7.4 | 7.93E-06 | 1.24E-02 | 1.22E+03 | this work |
| RAD16 | *rac*-methodol | 7.4 | n.d. | n.d. | n.d. | this work |
| RAD17 | *rac*-methodol | 7.4 | n.d. | n.d. | n.d. | this work |
| RAD18 | *rac*-methodol | 7.4 | n.d. | n.d. | n.d. | this work |
| RAD19 | *rac*-methodol | 7.4 | 3.55E-06 | 8.70E-03 | 5.46E+02 | this work |
| RAD20 | *rac*-methodol | 7.4 | 2.55E-05 | 2.57E-02 | 3.92E+03 | this work |
| RAD21 | *rac*-methodol | 7.4 | n.d. | n.d. | n.d. | this work |
| RAD22 | *rac*-methodol | 7.4 | 3.55E-04 | 6.01E-01 | 5.46E+04 | this work |
| RAD23 | *rac*-methodol | 7.4 | 6.50E-05 | 9.22E-02 | 1.00E+04 | this work |
| RAD24 | *rac*-methodol | 7.4 | 3.65E-04 | 1.76E+00 | 5.62E+04 | this work |
| RAD25 | *rac*-methodol | 7.4 | 4.50E-05 | 1.59E-01 | 6.92E+03 | this work |
| RAD26 | *rac*-methodol | 7.4 | 1.13E-03 | 1.67E+01 | 1.74E+05 | this work |
| RAD27 | *rac*-methodol | 7.4 | 2.44E-04 | 3.70E-01 | 3.75E+04 | this work |
| RAD28 | *rac*-methodol | 7.4 | n.d. | n.d. | n.d. | this work |
| RAD29 | *rac*-methodol | 7.4 | 3.14E-02 | 2.93E+02 | 4.83E+06 | this work |
| RAD30 | *rac*-methodol | 7.4 | 1.51E-02 | 7.16E+01 | 2.32E+06 | this work |
| RAD31 | *rac*-methodol | 7.4 | 1.79E-03 | 2.66E+00 | 2.75E+05 | this work |
| RAD32 | *rac*-methodol | 7.4 | 1.11E-02 | 7.82E+00 | 1.71E+06 | this work |
| RAD33 | *rac*-methodol | 7.4 | 2.22E-04 | 2.36E-01 | 3.42E+04 | this work |
| RAD34 | *rac*-methodol | 7.4 | 2.26E-03 | 1.19E+01 | 3.48E+05 | this work |
| RAD35 | *rac*-methodol | 7.4 | 3.66E-02 | 1.05E+02 | 5.63E+06 | this work |
| RAD36 | *rac*-methodol | 7.4 | 6.18E-05 | 1.14E-01 | 9.51E+03 | this work |
| **Computationally designed retro aldol enzymes (from existing scaffolds; no laboratory evolution)** | | | | | | |
| RA34 | *rac*-methodol | 7.5 | 1.22E-04 | 1.90E-01 | 1.90E+04 | ^22^ |
| RA45 | *rac*-methodol | 7.5 | 2.83E-05 | 3.60E-02 | 4.00E+03 | ^22^ |
| RA60 | *rac*-methodol | 7.5 | 1.55E-04 | 2.70E-01 | 2.40E+04 | ^22^ |
| RA95 | *rac*-methodol | 7.5 | 3.33E-05 | 5.30E-02 | 4.80E+03 | ^22^ |
| RA110 | *rac*-methodol | 7.5 | 8.33E-05 | 4.80E-02 | 1.20E+04 | ^22^ |
| cRA-50 | *rac*-methodol | - | 2.2E+00 | 1.8E+02 | - | ^76^ |
| RA117 | *rac*-methodol | 7.4 | 2.76E-05 | 5.8E-02 | 4270 | ^77^ |
| **Computationally designed retro aldol enzymes (from existing scaffolds; laboratory evolution)** | | | | | | |
| RA34.6 | *rac*-methodol | 7.5 | 3.67E-04 | 1.20E+01 | 5.50E+04 | ^22^ |
| RA45.2-10 | *rac*-methodol | 7.5 | 2.32E-04 | 5.40E-01 | 3.60E+04 | ^22^ |
| RA60.2 | *rac*-methodol | 7.5 | 1.17E-03 | 1.80E+00 | 1.80E+05 | ^22^ |
| RA95.4 | *rac*-methodol | 7.5 | 2.50E-04 | 8.40E-01 | 3.40E+04 | ^22^ |
| RA95.5-8F | *R*-methodol | 7.5 | 1.08E+01 | 3.4E+04 | 1.7E+09 | ^5^ |
| RA110.4-6 | *rac*-methodol | 7.5 | 3.83E-03 | 5.50E+01 | 5.90E+05 | ^22^ |
| RA95.5-8 | *rac*-methodol | 7.5 | 1.7E-01 | 8.50E+02 | 2.6E+07 | ^44^ |
| RA117.4 | *rac*-methodol | 7.4 | 2.1E-02 | 5.6E+01 | 3.23E+06 | ^77^ |
| RAβb-16.1 | *S*-methodol | 7.5 | 2.50E-02 | 1.08E+02 | 3.8E+06 | ^31^ |
| RAβb-16.2 | *S*-methodol | 7.5 | 2.67E-02 | 5.0E+02 | 4.1E+06 | ^31^ |

## Table S6. Amino acid sequences of MBH constructs.

All genes were cloned into a vector featuring a N-terminal hexa-histidine tag and a TEV cleavage site with the sequence MGSSHHHHHHSSGENLYFQSG. Extinction coefficients were calculated from these sequences using ProtParam^77^.

| **MBH** | **sequence** | **extinction coefficient [M^-1^ cm^-1^]** |
| --- | --- | --- |
| 1 | GEAARRAGETFGEVLNAGDEANPALAPLAEANRRFRREHADLVERIYALGAEAIERLGTADPAAARAVVAVMLGLLNVARLAAELRDEGKDEEADRLLDLAEEILRAALAGTPEEVITVANAVGQAAWLAYIAGKRADLALENLEKVRNANLEEQKAFAEAGAAASVAWLAATYGPEAAAAHLAAVARAVDAAVDVLRAA | 15470 |
| 2 | GEAARRAGEAFGEVLDAGDRANPALQPIADENRRFVRDHADLVERIYALAAEAIARLGTADPAAAAAVVAVMLGLLNVARLAAHLRDQGRDAEAEELLALAEEILRAALAGTPEEVTTTARAVGQAAWLAFVAGRSVELALENLRKVKEANLEERRAFAEEGEAASVRWLADTYGAEAAAAHLAAYAAGVAAAVAILQAS | 15470 |
| 3 | GQEFADAVAEAGRIIIRHDRASPHPALQAHAVFSAAFGERNGDTIAAEIVAGKTDFRAGFAAWNATLERVAALHPEYAPLLEKVIALNEEMTAHLDAAPVTSEEEAAMRAFNAAIGVGNRLRAADPALDTYISTEEGAEAALIGGVAMLLFRGKFAGPEYQAKAEEILAQLPPELQAAVHNHLAGLEEAFAHFKKVYLSL | 11460 |
| 4 | GQARADAVARAGEIIIRHDRASPHPALQVHAEYARAVADEHGDEIAARIVAGENDFVAGQQAWVDTLAQVAALHPELAPLLDQVAALKREMTAHLAAAPITSDDEAAARVFVADIGVANRIRAADPRLAAYIATREGAEAALIGGVAMLLFLARFAGPEFAARAEAILAQLPPELQAAVHQHLAGLEEAFAYFKEVYLSL | 11460 |
| 5 | GPPPPGPVSPEGQAARDGHLAGLEAGREVVREAVRAFLPEDPEAADLIADLAWALFQEYLARLRAQAETAEEALATIPLAFELAARGAVEIYNAVTGRLTPEEAAALVEVLREALRRGYAVLLAFDLSFSDEAYAEGMEAMRNDFAANYDAIMAALRAELEAETDPIRRATIQAEIDGREAWNANLDHITKLTIEGRKIL | 18450 |
| 6 | GPPPPGPVSPEGQAAADGHREGLEAGREVVREKVAAFLPEDPEAAEKIADLAWAKYLMYLAKLRAQAKTAEEALATIPLAFELAAKEAVDIYNAVTKRLTPEEAEELKKVLYEALKEGYAVLLAYDLSFSDEAYAEGMELLRRTFAANYDAIMAALKAELEAETDPIRKATIQAIIDGKAAWAANLDHITELTIEGRRIL | 22920 |
| 7 | GRLRRIVALSRKILEIHGAGLKQMPDSEFAALLADLALAYADYVQALVDGDDAAAAAAKARVDALTARINADPVGRAALETWAATLRKEVPAYAAALEEARAAEAAGDRAALAEALARALAYEAIAIVRNIENKDEVLAARRAAYEELVPKAVGRPELEVLAEMYRWLAEIAAHPKHRAIDDKYAEELKKLAEELERLLA | 21430 |
| 8 | GAAAAGYYVREGIAVVRGELEAELTPEELAAFDALFARIEARVEPLITAGDPPLALVRAAFYAANNTLRGLDPAILRLFGATDEEIAAAQAVARKLLVEFERALAERDRLTDEELTARLEAIAVEAWNAIAPSALRSPYPVLRAAALAGARTTPALSAEDAALRAAALAAATPEELAAADAHDAAFAAVDARAQELYDAA | 12950 |
| 9 | GAAAAGYYVREGIAVNRGELERELTPEQLAHWEALFEKIVAEVEPLITAGDPPLALVDAAFYAANNTLRGLDPGILRLFGATDAEIAAAQAVAERLLVEFREALARRDELTDEELTARLRAIAVEGWNAIAPAALRSPYPVLRAAALAGARRTPALSAEDAALRAAALAAATPEELAAADAHAAAHDAVDARARELYEAA | 18450 |
| 10 | GLRELLGRHLAGAALLDGAIVDPALVPQAVAFLQQPNPALSPVQRQAVIDALRALETGLTPEQRAGIRAYLEANVKERAADPARLEAAEALAIVLGQKSLGVTDIPADLVAKAEVARTAWDEETASYADVTDAIADKVVEIGTTRLGIDADLARSTGRYGGLFVALGILKDPELAVHIAIVHAASLILIGYAALIEQQLA | 11460 |
| 11 | SALLERAKAKAKELGEIWKSMLGPVFKKVFDADPERFFKANLGGLLYYVMVAERGAEAADAELRKISPEVAAGIEADRDILEGNPELRKEIIERGQEYIDRLKVEDPERLKKIEDLFEKILEIRREFLLIGGMSEEETDAFLEASRGFELLYVFFLLYIDRLLQEDPEKGLALVKKFAPYFVAHFNRFIILAEKYEAEAK | 15930 |
| 12 | GGKIWGIAHAGIISLDIGRAETTDPRLLEFIDLARELLRAVARAALAMPEEEVEKMWEFFKQVVPEERLTAAPPVPDVPLTEEEFEEYLNRAIELAEELARRFEGELLELLAEHGRRHASSLTPEELARVEKANKLILEELEKIRTEISKVGTPEQIATANRIIDFIRKLIETAPYSAIVWGTILGLLMLLWVLLWKAKQ | 30480 |
| 13 | GDAAVAGELREGAEKADGAIIGDYDIKEVAQELLDLAKRYGNPTLVKIAEIGLNNIDTEITPKGRENYRKIIETAKVERAANPELKEKVEKFQYLFGRAVLAPVEEIPALMAEAVKHSQWWLDDPFGRAYLALMINVVIKANPEKGRAIADAFGFDDADLAAGVAAMGALGITEAIAAIHLTGNVLAALAEDFAARAAAA | 18450 |
| 14 | GAALAGAEAAAGAVLAARHAARPEAVAAEKEIEAAPDMAAAVEAAARYFRAGLDDYAAEIRAEADSPAASPRASAFMRALADAMREGAALMAEDPIAGLYALILRVEAIIKADPEAAFEASDFHFSLPEYAAFRPVSERKAKEIAARVPEIVAEAGLPADVQAAMVYVAEVAAEAGGGPLWPFLEIIEALERAVAAVKAK | 12950 |
| 15 | GGAEFGAMIAGTGAAVDGLRALPGGEAAAFAVDVLGQNEILAYMVELAAGDPDIAAEFGILVVAREVAHIYGVLRDDPEAAERLGNAFLDVALGAKTVEELRAGVLAVYRALMEHLAKRDPSWLDRLALWEEVVDKVEPESYWTNFLALVNGALARYSREEIRARALELAREAVATGDADAAMDARLVAAVERAEARKAA | 23950 |
| 16 | GGLELLGAYARYGLEIAGKEVAADKERYEELSQLLVEVGAALDEALAALRAGDYERAVEIILDTLEKHREKLTYLGRRHEKYLLENKDDPKLAKDVASYENLKKSLQAIVDGDLDTAADYYRKSLEAVRDRYVALHPDGETVYQEFVDRITNTIRNANPETYAILKRVSEFLLEAGISGLLWALLEVLLYALSGVQEAAQ | 24870 |
| 17 | GGLYEFAGRFAAGAVERGKLWKTVTKATYHYGVDYVVGKALQLGREEPELAASYRALARGLIRERLYLDLYHAGRRDEAIEIIQGHYGVSREAAERILDRRVAEEKEYLASPPPTDEEVTAALRGLAGYRKRLTQAVLDRFGREDDVILVQLYLDLSSDPELLADFLSHSDAEIVASSRGIDDRLQPVYDARLAEIEAKL | 23380 |
| 18 | GVAEAAAGTRAYYGARHGVGLLEGILAAFPDLPAEDRAYLEDALAYYRGLAEKYQPVVDANPEVSAAILDFIAEQLLASLRARIESGENPVLELAAGVVRTLEDYARAVGKPEAIEVGKRVALLVAKTGAPTIWLSLGRADAELLPKYREEIIAGYQRLAELAVKYAREVTMEEIEAAIAKWEAAQPAIDAAFQAVVDSL | 25900 |
| 19 | GDGLARVEAAFGALADALAALAGEDPLAGVLAEWVRLIPPVLYRDDPALALALADLLAAAVADDPVAAARAFLDANRYIDAVDDEVAAKDPAARKAILDAGWAYLRGGGDRQPLLDAARAHPEAFRRHCLASVEASRRSVRRARADPARARAYTAVAVLMGVFGVVDAEALLEAGVGNQELVDEARGALDEARALLEELL | 16960 |
| 20 | GLRELLEELGAIERAGLANLVAHPERAAVIRGTDAANLARFRATGNEAFVELRKRFYLSIANAIKRAFGWSEEEMRTWLATAGADPALADRLPDDISDEQLAYFLSAELVVGCTYALGGDPADVRALGRRLFSDWLDFASDAPIVQELLSTFSPEEIELFIDGLVDHAVAGSVRFADEEAVRRALEVGERGVAWAKSLKA | 26470 |
| 21 | GMEKVEELSAESVRVTATELVRLAREDPAAARAHAEAGKVVGKKVYEACQELAARSTSERVRTLLRFIGENELIGAEMDEMALAGASVEELVAFAEERSAEIAAPLEGWPEGFLLTFNIAFNTGWLAGVTGDEELYNKALEYFEKVGIPREDAEKILDIRLEMAKADAADGSPVLAAAIARITEVTLKLIKAEEEALNSL | 15470 |
| 22 | SPLAALLREVAAAILGGDPERGLQLAKKLWEGLWWLGVDPARGEEILAEVKAATGLTAEIDTLAAGVRELTRTVIEADERLRAEDPAAYAAIMAFAKLITTLKLYPEEVSPEEAKAAELFLKGEELIEVYLKGGPESYLTQGKKYVGEAAKILGLDPEVAWRGAVIHAEGVVKMTPEEAAALAAMDATLEEAIAAAEARL | 29450 |
| 23 | GMEEVERLSEELVRVTATELVRLAREDPAAARAHAEAGEVIGKAVHDACKELAAQSDSERVKTLLNFIGENELIGARMDRMALAGASVEELVDFAKKNNAKIAKPLEGWPKGFLLTFLIAFYSGWLAGVTGDEKLYNLALEYFEKVGIPREWAETILDIRLGMAKADAADGSPVLAAAIARITAVTLELIKACEKALNS | 21095 |
| 24 | SPAAALARELLDAILGGDPERGLALAKKLWEGLWWLGFDPARGDAILAEVKAATGRTAEIDTLAAVVRELNRTQVAADEELKKADPKAYAAIKAFADLVTTARLYPEKVTPEEREAAELFLRGERKIREYLRGGPEELLEQGRVDVARAAEILGLDPDVAWRGAVVHAQGSVAQTPEEAAALAELDATMAAAIAAAEAR | 26470 |
| 25 | GEGLARVEAAYGALADALAALASRDPRAGVLAEWIRLQPPVLYKMDPALALKLAELTARAVARDPEAAARAFLDANRKIDAVDAEEAAKDPAATADILAAGWAYLRGGGDMQPLLEAARAHPEAFRRHCLASVEASRESVRRARADPERALAYTSVAVLMGVNGVVDALALLLAGVGDQGLVDLARGALDEAEALLDELL | 16960 |
| 26 | SAAAALLQRLLDKILGGDPVEGLKLAKKLYEGLWWIGFDPARGNKILDEVRKATGLDAEINALAAVRRKLNATVIAADEALRAADPAAYAAIRAFADLVTTLKLYPEKVTPEQAEAANLFLAGEDLIAQYLRGGPESLKTKGVEYVARAAKILGLDPAHAIRGAFVHVQGLVDRTPEDRAALAAMDATASAAIAAAEARL | 18450 |
| 27 | DPAHTALEAAYRAVVGVLGEAVVDAFLKATALAEIAGFRLLLEGADLETALAAAREAVRAYLPTLVEATGNRAVAEAIAEAAFAHAELGLRVAEVLRADPALFRAYLEDALKVNEAMVEDPRKGLRLFVDFSKKYGKLWLIAVGKLHGAKTDEEALKLGKKYSPLVDLRGALRIFEAYAAATGRGAELAAATRAEIERLE | 14440 |
| 28 | MKAVEEAEKRYEEIVSVLGEAVVDALWKATALAEIAGYEALLAGADLAEALALARQAVEDYLPTLVEATGDRAVAEAIAEAAFAHAELGLRVAEVLRADPALFRAYLEDALKVNRAMVADPREGLRLFVEFSKKYGKEWLIAVGKLHGAKTDEEALKLGKIYSPLVDLVALYKVFEAYAKATGKGKELAEETKKKIEETL | 22920 |
| 29 | MEELEKYLAALAERKKAVDARVAERLAKIDPAEWARAAAPIGLRLADLVEELGFPQEVVDAMRADPAVAGDVLSRAVVEALAERVRQELAAQGLAELGEIVANLLITEADVLRQELALEFEDMYHAGATLDDLAARADELAAALRAAAPSELVGLILEAAVLGGLLHALMGIDIELGGKAWPYIERTVRRLYELYQVATA | 18450 |
| 30 | GEALRARALELGRELGEAFRRSISKLTPAEIQAHADAGAALAAVPVDPEAFLAAARAYMRVDAEVGERAGLCDSADELFARGLENVELALGGVKELGLESRLPELVAELVALTKAGAAKDKELADDTAAYADYVRDLVVGLVQAVVDASRTLPAEEAVFLAALVALWIAIAATLSDEVVEAGRRVIEALLDLARYLKAAS | 11460 |
| 31 | DLRELARAVIRALDAVHQALIATSDEGAQYVRDWYATLLAALDRLGLGDVRDEVLAAIERHGLAAILDVVLGTLTPEQIDKAVELGPVHAETGLALQEAVFGGASPEEVAAAKEKVAAADAEVMAQLRPLITEEQVEAVAEALGVSRRAALALILLTLVIYGGTVAEVALLVEGTPEQKARVAGLLAQLAAEYAALLARL | 11460 |
| 32 | GDGPAGLLAALAEMRAAGASPEELFFFACSVILEALQTDPVLGQAMLDALATLPEEVQEIALEVSRLHLEGTLVHQDELDAARAAVAAATAALLAALAAAGADAAQLAAARERLAQAVTGAWFRGAGYPHVAARYQPYLDWARAADPALAAALDALVAVGEKIAEFDVEAMKLVRKITDEELLRRATETLVAALRRVAAQ | 15470 |
| 33 | GEELRERALVLGRELGQAWRDSVLQLTPEEIRAHAEAGAALAAVPVDPAAFRAAARAFMRVDAEVGERAGLCDSAEELFARGLENVDLALAGVDELGLAARLPELVAELQRLTRAGAATDFELADDTAAYAETVERSVVSLVQSVVDASRELPAEEAVFLAALVALWIAYAATISDAVVEAGRRTIEALLELARYLKAAS | 15470 |
| 34 | PVDAAGVLAALDEMRAAGASPRDLFLFAAGVVLEFLLYDPAAGRAMLEALAGQPAEIQKLALQVSEMHLAGSLKYKEELAAALARVEAAAAALLARMRAEGASDAELAAARRLLADARRAAWYRGAGYDFVAERYQPALDWARAADPALGAALDEYEAVGKKIAKYDVEAQKLVDAITDEAALTAATEALRAALRRVAAA | 21430 |
| 35 | GADELAQRLGELIRGGLANLKAHPERAAVIRDTDAQNAARFRATGDQRFADLVDRFYLAIANAIIRTFGMTDEEAETWFRLGGGDPADLSKFEDTISRDHLLYFLSIELVVGCTHALGGDPKDVRALGRKLFKDFLRYASDAPIVQELLEGFTPEQMELFLDALVDHAVAGSVRFADEAQVREALEVGREGVAWAKRRAA | 15470 |
| 36 | SPELAEKLKKKAEEMRAWLEEHAAEVRRTTLAVERALARELLAEARAAGDARAAAVWEAYQAVLAWLSDPANYALWLRVAAANPRLVVSRAVDDLAVGVLAVICLDMTREEIEENKEELIAIYAVLHATQSIAVLETVIATPELADRWVGMLDLSPEERARALVDIWAEASNSEEGKKRITEAINKVLKEIAKELEAKKK | 37470 |
| 37 | GPGFTPEEQALVEELAKKGEELTKAIFEAASKHERAYYEAQGKPELYELILAHGERALKDGAARVAGLGDRLLTAEGQIELGRAYVKAAPMDFLHFKDAIETLGLEDKIFNGNLHEFLTALLKAVDYMLKNADVYGKRHAEQGVAATERMIEEGVDLTDYDAFRAAWERLNKEVFPEEYEEALTYFKKLEEVIRRLHAEL | 18910 |
| 38 | GQESVDFGRRGLELLAEKKLYKSLAAYYAALHIELLLLPLALSVLPIPQEAVEAFLGIYRAAQEILAKNPEAAKLVPDMIPYGLAHVAGWAGTTAINVQIAQEAQLTSPEKRIKYLKAWLNGFIAGFVSPEEAEKLIKEGAELLSEIVPKELAERYYEASLVGAAADQQATDGILTPEERKATAECFEKAVEEVIAKIKA | 22920 |
| 39 | GVEEAARRLLDIYLEALQVTVEHADEFIKYLPIHVKGSEEGTKLYAAGDYVGATRTIISADREIAEIIGNEEAVKLFDELLAEAERRAALRRELYAEAGLTDADVVAILQAGVDYDRSLLTYSPEEAAALLRKETQAEIDALRARLYGDRPLTPEELRQRAAFVAAWLLGGGTAAAIFDPKLYRRMLDAALAFHAALKAS | 18910 |
| 40 | CPGDTPEEAALRRELAARGAELTRAVNDAAFKYERAYYEEKGEPERYERLKRFGERALAQGAARVAALAPELCTAEGRVAFGKDYVVASPVDVLNLKDAIETLGLEEEIENGSKEQFLRALLRAVDYMLEHADEYGKKHAEAGVAATEEAVRRGVDLDDFDAFSAAWKELNKEVFPEEYKEALEFWAKIDEVIRRLAAER | 23045 |
| 41 | MKELLEKLKKRTEEMKEFLKKHEEEVRKKTFEVERKLAKELLAVAKAAGDARAAAVWEAYIKRLDWLADPENLALFERVAAAEPRLVVSRAVDDLAVGVLAVITLDMTKEEIEENKDEIIEIYAKIHAAKSVAVLDTVVATPELAERWVGNLDLTPEERAREYVAIWAEASNSPEGRRRIAERIGALLVEIAKKLAAKLL | 26470 |
| 42 | GEASVRFGRLALQNLGKAKLYKAMAATYAALLVERALLPFALRVLPIPEEAVAAYRGIYDAAEEILAKYPEAAKLVPDMIPYGLAHVAGWAETSVLNVRIAREAKLTTVEKRIAYLRAWWNGYGAGFVDPETAAKMIREGAKLLSKIVPKELADEYEKAAIKGAGADQEAGAKILTPEERAATHACFAAAVDEVIAELKK | 29910 |
| 43 | SREEVARLAGRALELDVRLVEDLRAADPEKAEAWLEGARVPGPPVSNVTRLANAALGMLFEAAYVKLKVGGISMEEALDRLYRLQLDWALAASVEELIALGREAGKADLAACYRADQILAADPSIIPDPARAEAIAARALKGEGIDEGVALLATLPADVLIELGFTHARVGLERNYGANEEELEKARELGTAMIRELTAP | 16960 |
| 44 | SPALAAALAALAALKKRVDARVAERLAKIDPAEWARAAAPIGRRLARLVAELGFPQEVVDAMLADPARAADVLGEAVIRALAEAVRRRLAALGLAELGEVVAAFLVEEARVLAQELALLFEDAYHAGASLDDLYARAEELAAALRAAAPDELTGLIRRAAVLSGLLHALVGIDVEEGGKAWPYIERTVRELYRLYQIATA | 18450 |
| 45 | DDAAAQAVIDAILTEGGALWAKYPELWEAVLDAHALAGALLDRAAAKTGKPLLDITPEEETPELREAKAILSGTDIRVDRLIEKYLPGKAFLFRGPLYLAGSVKAIELGKLREWLGNILDAYVPELRALGLDDADLEALTAELVRLFTLAAQDPRVLALSKEILEKEEAIQAEAAGLTDEEIRARLEEALARVEEILSKI | 22460 |
| 46 | PPLPPFGPPELEERVREELERAREVGIKVGNAVEGEMVARGGYTEEEVDKLVKEGADLIRNATSVEEIGIGMGMIHAGATGYAYLLLPEEKRPGLLALTAFGIAATAAVGPEAAAALMRAAFPETVAAWEEVAPAVLDKVLETIGVDADTAAVLREIDAATRGRGVDVLIEALARQALLRLHGVEVSLKEVVEALKRELL | 9970 |
| 47 | MKERVARLAALALERRHAMVARLAAADPAKAAAWAAGAAVPGPAVSATTRLANAALGNLFEAAYIKLKVGGITMEEALDIHFDELLKWALAASLEELIAIGREAGRADREACARADRILAADPSIIPDPARAEAIAARALRGEGIAEGVALLSTLPAEVLIELAFTHAKVGLERIYGANEEELKKARELGTEMIKTLTAP | 13980 |
| 48 | GAGAALGRRLLGAILGGDPREGLALAKRLYEGLWMLGVDPERGEAILAEVRAATGLDAEIDALAAVMRALNATIIAADEELRAADPAAYAAVLTFADLVLTSKLYPEKVSPEEKKAAELFLKGEELIAEYLRGGPESYLTKGKEYVAEAAKILGLDPAQAWRGAVVHVAGSVARTPEQAAALAALDRTFEEALAAAEARL | 19940 |
| 49 | GLGELLGQLGAILRAGLANLVAHPERAAVIRDTDAANLARLRATGNQRFVDLVQRFYLSVFNAMLRAFGWTREEGEVWLAVAGLDPALLDRLPDTISDENLAYFLSVELVVGCTHALGGDPEEVRALGRRLFRDWLEFASDAPIVQELLATFTPEELELFLDGQVDHAVAGSVAFPDRAAVEAALRVGEAGVAYARSLAA | 20970 |
| 50 | GVGEAGRRLLGLYLKGLEVTIEHADEYIKYLPIHVEGAEKGTALYEAGDYVGATRTIIEADRKIAELIGNEEAVALFDELLAAAAERAELRRALYAEAGLDDADVVAILRAGVAYDRGLLTLSPEEAAAALRADTQAEIDTLRARLEGERPLSPEELRRLAAFVAAWILGGGAATAIFDPELYREMLAAARAFYEALQRA | 18910 |
| 51 | GAGLAARLGARGARMGAWLEEHRDEVLAKTFEVERKLARELLAEAEAAGDARAAAVWRAYLEVLDWLADPENLALWERVAAATPDLVVSRAVDALAVGVLAVICLDMSDEEIEERKDELIRIYAIIHAAQAVAVLETVVATPELADRWVGMLSLSPEERAREIVRIWAEASNSPEGRRRIGERILAILKEIAEKLEAEKK | 35980 |
| 52 | GLGEAALGVIRGLDAGHRTLIETSEEGAQYVDDWYAVLLRALDRLGLGAVNDEVLAAIKRHGLYAITEVVLGTLSPEQIQKAVDLGPLHAEAGLKLLEAVHGGASPEEVAAAQDAVADVDAEVMAQLRPLISEAQVEAVAEALGVSREAALALILLTLAIYGGTVAEVRLLVEGTPEQRARVAGLLAQSAAALAALLAR | 11460 |
| 53 | GAGLAGGAAAAAGLAGALAAIYSTPEGKLVHRLWLTGAVLARDEVLAVADEAAAFARRAVDEKLDDETIAREAGELHARAAVVILRAVGLDAARLAAYRRVQAAMIAALLAAADAYAAAGLDFRALYLRAVVEAELAREPELDRIPVGDAPLLPITPEAEAIAFAADRYIDALIDYRVAARVDPALGEAARAALLALVDA | 14440 |
| 54 | GAGEGVRRRLGLLVVGARGYARLTPEEQRLIGVHGIAGMKIIEEKGLPDTEEEWIETGGTRELYEAWKATLARLLAALPPPDELDEAAVERYLAILSEFLREQAPLIVGGDEKAGYPHAELEAELAADPAAWRARVERLFARADEEFRLGTPEAFERAIKYFLTGLEAGLGVAGLAGPEAVAALCARAAERAQAWLDSLR | 29450 |
| 55 | AARAAGFDAIAATPEGRRRFFELLLRLPLVYDLLMAELLLARTEGASPEEQAELRAAADNLRAHFARSTSDPAAAEAAVLPLVDLLLEILAPYRDTPEFDEAVEAALTIARTAPAFDAATPAAVRALRVAAGDAFTAAARALNAGDLDTARVHVDAGLALAAQSWAANRAAGLGPQVDTLLAASKVISAFVRKAVEEYLA | 9970 |
| 56 | SAAALAAAAAGVAALGERRAAIVAAYRGGYAADMKIFGFTEAEVRELAERIRRPPFTGFTPEQLGQILLGTAAAQDAWDKVVEAAGSYEAVARAYWARRVEEVGADEEEARQAGLIHAALSLVAAELYATLSPEEAVLTLLSTRYYACLDALKEYGKKVFLLLSGDFLTAGAARLLVEDPSLNLDEATARTREIIESIRK | 22920 |
| 57 | ADLATGLERINATPEGRRRFFELLLRLPLVYDLLMAALLLRRTEGASPEERARLTAAADNLLAHFARSTSDPERARAAVQPLVDVLLAILKPYEDTPEMDKAIEAALTIADTAPAFDAATPASVRALRVAAGDAFTAAAEALNAGDLDTAEVHVRAGLALAAQSWAANRAAGLQPQVDTLLAAVEVITAFVRKAVEDYLA | 9970 |
| 58 | GPGKEIGEKLLELYEETVRLMRAEPGDPRSICLYDIVFHSLLIVLDPRLAPITEPLIALNQAALDAGTAEAAAAAHIETSKRLPEIYRAAVDDPATSEDALRFLIEFTYYWAKVGSSLPSARALVEALAPVIKKIDAGTATREELRAALGAALPLIEARIAEGVAADEAATPEEVCLGFDKVFLAVLELRDVLREYIENL | 14565 |
| 59 | SEEARRRREEGIRRLEERREEIVASYLGGLKADMAIFGFTDEEVRALAEKIRRPPFTGFTPEQLGQILLSEADAFEEWKKVVEKYGSYEAVARAYWAKKVKEVGANEEELRQAGLVHAALSLVAAELFATLSPEEAVLTLLSTRYYACLDALRKYGKKVFLLLSDDFLRAGAARLLVEDPSLNLEEARARTQEIIDAIRK | 21430 |
| 60 | GEGKKIGEELLERYEEMVRLMREQPGHPASITMYDIVFNRLLTVLDPRLAPITEPLIAENEAALEAGTAEAAAAAHIANSRRLPDIYRAAVDDPATSERALEFLIRFTYYWAKVASALPAARALVEALAPVIAKIDAGTATREELRAALAAALPLIEAVIAEGVAADEAATPAEVCAGFDRTFLAVLEERDVLREFIENL | 12950 |
| 61 | AAALAAEIGAAAAEFAQLSLDEQIAAAIAKQQAWLDFVNSHTYEEIKALAAELAKDPRAAALVDLLRLAEVLARAVAARVRDGEAGRERLRKVLETATQYVIENSKPIHDKLVAEESIFPNYADFQRYRLEKELAILDAPTAEAAVDAVLDAIRDSLKVYKGPFHRTGLEYLVAHEAEVRAFLRAVADAAAAATRARLPR | 14440 |
| 62 | ALAALLARAAGIAALGAARAAIVAAYRGGLAADMAIFGFTEAEVEALARRIRRPPFTGFTPEQLGQILLGTAAFQAAWQALVDAAGSYEAVARAYWERKVAEVGANEEELRQAALIHAALSLVAAELFATLTPEEAVLTLLSTRYYACLDALKEYGPKVFLLLSDDFLRAGAARLLVEDPSLNLEEATARTREIIEAIRK | 19940 |
| 63 | SPALEAALEAAGPRAAAAIERLRPLFDYLVTISKADIDPNLPAEERERIAKAIDDFFYGRPGFALAAQGVWFRHMAEITPEGPTREAYRAGAEGVLRILRGEDLEAGLEGLRRGLEEVVRVNAKEEALEPELRKELETVTGEEVVARAERLFAEGRIVEAGATLHTGAQHPKTAPVASKWLRDRNAALLERGFAELAGVF | 15470 |

## Table S7: Conversion and design parameters for MBH designs

Conversion ± standard deviation of substrates 2-cyclohexen-1-one (**3**) and 4-nitrobenzaldehyde (**4**) after 8 hours with 2 mol% of enzyme or small molecule catalyst. Designs with identical backbone numbers have the same fold. Relative conversions denote conversions relative to the construct with maximum conversion, MBH48. AS model indicates the active site model the design was based on. Fragment picking indicates if an idealized helical backbone fragment was used as scaffold (no) or if fragments were picked from the PDB (yes). CM refinement indicates if the Rosetta Coupled Moves protocol was used to generate final active site sequences instead of LigandMPNN.

| **MBH** | **backbone number** | **conversion [%]** | **relative conversion [%]** | **AS model** | **fragment picking** | **CM refinement** |
| --- | --- | --- | --- | --- | --- | --- |
| 1 | 1 | 2.68 ± 0.57 | 16.77 | BH32.14 | no | no |
| 2 | 1 | 1.14 ± 0.2 | 7.12 |  |  |  |
| 3 | 2 | 1.80 ± 0.4 | 11.25 |  |  |  |
| 4 | 2 | 0.77 ± 0.18 | 4.84 |  |  |  |
| 5 | 3 | 1.32 ± 0.24 | 8.26 |  |  |  |
| 6 | 3 | 2.67 ± 0.69 | 16.71 |  |  |  |
| 7 | 4 | 1.00 ± 0.17 | 6.28 |  |  |  |
| 8 | 5 | 1.05 ± 0.14 | 6.57 |  |  |  |
| 9 | 5 | 1.46 ± 0.22 | 9.15 |  |  |  |
| 10 | 6 | 1.88 ± 0.23 | 11.76 |  |  |  |
| 11 | 7 | 1.56 ± 0.11 | 9.73 |  |  |  |
| 12 | 8 | 1.52 ± 0.2 | 9.52 |  |  |  |
| 13 | 9 | 1.43 ± 0.15 | 8.95 |  |  |  |
| 14 | 10 | 1.81 ± 0.3 | 11.33 |  |  |  |
| 15 | 11 | 2.4 ± 0.39 | 14.98 |  |  |  |
| 16 | 12 | n. d. | n. d. |  |  |  |
| 17 | 13 | 1.32 ± 0.15 | 8.25 |  |  |  |
| 18 | 14 | 3.01 ± 0.48 | 18.83 |  |  |  |
| 19 | 15 | 4.78 ± 0.65 | 29.91 | BH1.8 | yes |  |
| 20 | 16 | 1.51 ± 0.22 | 9.45 |  |  |  |
| 21 | 17 | 1.12 ± 0.04 | 6.98 |  |  |  |
| 22 | 18 | 2.17 ± 0.23 | 13.55 |  |  |  |
| 23 | 17 | 0.53 ± 0.01 | 3.33 |  |  |  |
| 24 | 18 | 2.1 ± 0.16 | 13.15 |  |  |  |
| 25 | 15 | 0.61 ± 0.12 | 3.8 |  |  |  |
| 26 | 18 | 5.31 ± 0.82 | 33.18 |  |  |  |
| 27 | 19 | 1.7 ± 0.12 | 10.63 |  |  |  |
| 28 | 19 | 1.43 ± 0.04 | 8.95 |  |  |  |
| 29 | 20 | 4.4 ± 0.42 | 27.51 |  |  |  |
| 30 | 21 | 0.92 ± 0.16 | 5.78 |  |  |  |
| 31 | 22 | 2.82 ± 0.14 | 17.6 |  |  |  |
| 32 | 23 | 1.6 ± 0.14 | 10.03 |  |  |  |
| 33 | 21 | 1.26 ± 0.03 | 7.9 |  |  |  |
| 34 | 23 | 1.58 ± 0.05 | 9.85 |  |  |  |
| 35 | 16 | 1.67 ± 0.13 | 10.45 |  |  |  |
| 36 | 24 | 4.11 ± 0.16 | 25.68 |  |  |  |
| 37 | 25 | 3.41 ± 0.07 | 21.33 |  |  |  |
| 38 | 26 | 1.05 ± 0.09 | 6.55 |  |  |  |
| 39 | 27 | 1.62 ± 0.12 | 10.13 |  |  |  |
| 40 | 25 | 1.27 ± 0.03 | 7.95 |  |  |  |
| 41 | 24 | 2.05 ± 0.1 | 12.83 |  |  |  |
| 42 | 26 | n. d. | n. d. |  |  |  |
| 43 | 28 | 1.9 ± 0.02 | 11.85 |  |  |  |
| 44 | 20 | 3.51 ± 0.22 | 21.93 |  |  |  |
| 45 | 29 | 4.07 ± 0.19 | 25.43 |  |  |  |
| 46 | 30 | 7.06 ± 0.38 | 44.16 |  |  |  |
| 47 | 28 | 9.46 ± 0.68 | 59.16 |  |  |  |
| 48 | 18 | 16 ± 0.57 | 100 |  |  | yes |
| 49 | 16 | 2.34 ± 0.47 | 14.63 |  |  |  |
| 50 | 27 | 1.78 ± 0.1 | 11.13 |  |  |  |
| 51 | 24 | 1.47 ± 0.05 | 9.18 |  |  |  |
| 52 | 22 | 0.34 ± 0.07 | 2.1 |  |  |  |
| 53 | 31 | 1.19 ± 0.06 | 7.43 |  |  |  |
| 54 | 32 | 3.18 ± 0.06 | 19.9 |  |  |  |
| 55 | 33 | 3.22 ± 0.95 | 20.13 |  | no | no |
| 56 | 34 | 10.94 ± 0.89 | 68.37 |  |  |  |
| 57 | 33 | 5.83 ± 0.74 | 36.43 |  |  |  |
| 58 | 35 | 0.55 ± 0.12 | 3.45 |  |  |  |
| 59 | 34 | 1.8 ± 0.1 | 11.28 |  |  |  |
| 60 | 35 | n. d. | n. d. |  |  |  |
| 61 | 36 | 4.86 ± 0.69 | 30.41 |  |  |  |
| 62 | 34 | 2.86 ± 0.14 | 17.9 |  |  |  |
| 63 | 37 | n. d. | n. d. |  |  |  |
| lysozyme | n.a. | 0.26 ± 0.09 | 0 | n.a. | n.a. | n.a. |
| DMAP | n.a. | 0.18 ± 0.02 | 0 | n.a. | n.a. | n.a. |
| imidazole | n.a. | 0.14 ± 0.02 | 0 | n.a. | n.a. | n.a. |

## Table S8. Comparison of designed and evolved MBHases.

Comparison of designed and evolved enzymes for the Morita-Baylis-Hillman reaction of 2-cyclohexen-1-one (**3**) and 4-nitrobenzaldehyde (**4**) according to a random-order fit. ± indicates 95% confidence interval of the fit. We suspect that high apparent Michaelis constants of both MBH18 (*K*_M_ of 8.4 mM and 6.2 mM for **3** and **4**, respectively) and MBH48 (*K*_M_ of 25.2 mM and 1.4 mM for **3** and **4**, respectively) are likely due to the challenging bimolecular nature of the MBH reaction, complicating the design of binding pockets with high affinity for two substrates. This is also reflected in the apparent Michaelis constants of the evolved variants BH32.14 (*K*_M,_ **_3_**: 2.56 mM, *K*_M,_ **_4_**: 1.14 mM) and BH1.8 (*K*_M,_ **_3_**: 12.02 mM, *K*_M,_ **_4_**: 323 µM).

|  | ***k*_cat_ [min^-1^]** | ***K*_M, cyclohexenone_ [mM]** | ***K*_M, 4-nitrobenzaldehyde_ [mM]** | **Ref.** |
| --- | --- | --- | --- | --- |
| **Computationally designed MBH enzymes (this work)** | | | | |
| MBH18 | 7.70E-03 ± 1.9E-03 | 8.33 ± 2.97 | 8.55 ± 3.73 | this work |
| MBH48 | 2.49E-02 ± 5.9E-03 | 25.16 ± 10.6 | 1.42 ± 0.35 | this work |
| **Computationally designed MBH enzymes (from existing scaffolds; no laboratory evolution)** | | | | |
| BH32 | 2.23E-03 | 7.998 | 1.77 | ^2,4^ |
| **Computationally designed MBH enzymes (from existing scaffolds; laboratory evolution)** | | | | |
| BH32.8 | 1.68E-02 | 4.173 | 2.381 | ^4^ |
| BH32.12 | 1.00E-01 | 9.719 | 0.893 | ^4^ |
| BH32.14 | 3.49E-01 | 2.556 | 1.137 | ^4^ |
| BH1.8 | 4.50E+00 | 12.02 | 0.323 | ^30^ |
| BH1.8 23H | 1.13E+00 | 12.19 | 0.293 | ^30^ |

## Table S9. Primers used for generation of RAD variants.

| **RAD** | **variant** | **forward primer** | **reverse primer** | **Ta [°C]** |
| --- | --- | --- | --- | --- |
| 1 | K123A | TGGTGTAGGTgcgTATGAGGGTATG | AGGAAAAAAGCAAGTGAG | 58 |
| 2 | K122A | GGGAATCGGAgcgGCGAAAGGAG | AAGAAGAACGCCAAAC | 57 |
| 3 | K119A | TGGCGGAATTgcgGGTGTTGCTG | ACGGCGATCGTCAATG | 63 |
| 4 | K119A | ATTGGGGATTgcgGGAGTTGCTG | ACCGCGATAGCCAATG | 60 |
| 5 | K119A | TTCAGGAATTgcgGGCGTAGCTG | ACGGCAATGGTTAAAG | 57 |
| 6 | K119A | AGGAGCCGTAgcgGGGCACGCGG | AAACCGATGGTCAGAGCTAATTC | 66 |
| 7 | K177A | CTCCATCATCgcgGGTTTGGCGTTTG | AAGCCTAACACCAATGC | 61 |
| 8 | K22A | CTTCGGCGCTgcgGGACTGCTTAATC | ATAGCCAGCAGGCGT | 64 |
| 9 | K65A | TGCTGCCGCTgcgGGGCTGGTTT | ATGATGGTCTCATCCGATAATTTCC | 66 |
| 10 | K65A | CGCAGCGGCAgcgGGTTTAACTTAC | ATAATAGCATCATCGCTAAGTTTC | 62 |
| 11 | K65A | CGCGGCAGCCgcgGGCTTGGCGT | ATAATTGCGTTGTAGCTTAACTTCCC | 68 |
| 12 | K65A | TGCCGCCGCAgcgGGGCTTGTAT | ATAATTGTTTCGCGCGAC | 62 |
| 13 | K65A | TGCCGCGGCTgcgGGTCTTGCTT | ATAATCGCGTCATCGGAAAG | 64 |
| 14 | K70A | TGCGCTTGTTgcgGGGATCGCTC | GCCAGCACCAGTGCT | 66 |
| 15 | K70A | TGCTTTGGTGgcgGGTATGGCCC | GCTAATACTAACGCGGC | 63 |
| 16 | K70A | AGTTCTGGTGgcgGCCATGGCATTATA | GCCAGGACAAGAGCG | 64 |
| 17 | K81A | GTCCCTGGTCgcgGCTACGGCCA | AGAAGTACAAGAATCTTTTTTACGACTTC | 64 |
| 18 | K81A | GGCCCTTATCgcgGCCACCGCAATTTAC | AAAAGGATTAAAATCTCCTCGATC | 61 |
| 19 | K81A | GGCGTTAGTAgcgGCTACAACTATCTAC | AGCAGTACCAGCAAG | 59 |
| 20 | K81A | AGCATACATCgcgGCAGCGACGATC | AACAAAATCAACATCTTCTTTG | 57 |
| 21 | K81A | AGCATACATTgcgGCAACCGCCATTAAG | AAAAGGATTAACATCTTGCG | 59 |
| 22 | K122A | AGTAATCGCGgcgGGGGCGTATTTTGATC | GTCAGGGCAGCGGTG | 65 |
| 23 | K122A | AGTGATCGCTgcgGGCTCTTACAAG | GTGATCGCATTAGTCAC | 58 |
| 24 | K122A | TGTTATCGCAgcgGGTGCCGTGT | GTTAAAGCGTTCGTTACC | 60 |
| 25 | K62A | CGCCACACTTgcgGGGGTGCTTG | GTGATGACGGTAAAGAATC | 59 |
| 26 | K76A | TGTCGGGGCGgcgGGATACGTCG | ATAACAGCGAATGCGGC | 65 |
| 27 | K62A | ACTTTCGTTGgcgGGGGTTCTTGCAG | GTGAACACTGTAAAGAACTCATC | 62 |
| 28 | K62A | ATACACCCTGgcgGGGGTGCTTG | GTAATTACAGTAAAGAACTCCC | 60 |
| 29 | N178A | GCGTAAGTTCgcgGAGGAAGCTGG | ATTTCCTCCACCTGTTC | 58 |
| 29 | K76M | TGTCTACGCTatgGGCTTAGCAC | ATGACAGCAAACGCGG | 64 |
| 29 | Y23A | TGCTCAGCCGgcgTATGATTTTGTCAAAAAG | TTCTCCTTCGCAGCTTC | 58 |
| 29 | Y124A | TCCGAAAAAAgcgTTTGGCGCAATCAC | TCCTTGATCAACTTCTTG | 56 |
| 29 | K76A | TGTCTACGCTgcgGGCTTAGCAC | ATGACAGCAAACGCG | 61 |
| 30 | K76A | TGTTGGAGCGgcgGGATTTGCAG | ATAACAGCGAAAGCC | 57 |
| 31 | K62A | CATGTTGTTGgcgGGTCTTTTCTATCAC | GCCAATACAAGCAAGTC | 57 |
| 32 | K62A | CATGCTGTTAgcgGGTATCTTTTATCACG | GCTAATACCTCCAAGTC | 57 |
| 32 | K62M | CATGCTGTTAatgGGTATCTTTTATCAC | GCTAATACCTCCAAGTC | 56 |
| 32 | Y120F | TGCACCGCAAtttTTAACCCTTATC | AATTCCTCCCAGGAAATAC | 60 |
| 32 | Y99F | GTCGCTTTTGtttGCGGAACTTC | ATAAGACGCTGTTCCAAG | 60 |
| 32 | N178A | AGAACCGATCgcgAAAAAGATGGGAGAATAC | ACCTCTTTGGTCGCTTC | 61 |
| 34 | K69A | AATCGCAATCgcgGGCGCTGTCTTCTCC | GCCAACGCGATGCCA | 65 |
| 35 | K62A | CATGTTTGTCgcgGGGATCTTTTTTCATG | GCCAGAACGTCCATATC | 56 |
| 35 | K62M | CATGTTTGTCatgGGGATCTTTTTTCATG | GCCAGAACGTCCATATC | 58 |
| 35 | Y23F | TGTAAAGGAAtttGTGGAGCGTG | ATTTTTTCACGATTTTTGATAGC | 58 |
| 35 | Y120F | TTGGCCGTTAtttTTATCCCTTATC | AACTCTTCACGGCTG | 59 |
| 35 | N178A | TGCACCTATCgcgGAAGAAGCTGC | ACTTCTTCGGTGGCC | 58 |
| 36 | K69A | AATCGGCATGgcgGGCCCGATCGAG | GCTAAAGCAATTCCTAAGGCTTT | 65 |

# Supplementary References

69. Dauparas, J. *et al.* Robust deep learning–based protein sequence design using ProteinMPNN. *Science (80-. ).* **378**, 49–56 (2022).

70. McPartlon, M. & Xu, J. An end-to-end deep learning method for protein side-chain packing and inverse folding. *Proc. Natl. Acad. Sci.* **120**, 1–9 (2023).

71. Abramson, J. *et al.* Accurate structure prediction of biomolecular interactions with AlphaFold 3. *Nature* **630**, 493–500 (2024).

72. Bannwarth, C. *et al.* Extended tight-binding quantum chemistry methods. *Wiley Interdiscip. Rev. Comput. Mol. Sci.* **11**, 1–49 (2021).

73. Pracht, P. *et al.* CREST—A program for the exploration of low-energy molecular chemical space. *J. Chem. Phys.* **160**, (2024).

74. Olsson, M. H. M., SØndergaard, C. R., Rostkowski, M. & Jensen, J. H. PROPKA3: Consistent treatment of internal and surface residues in empirical p K a predictions. *J. Chem. Theory Comput.* **7**, 525–537 (2011).

75. Elaily, W. *et al.* Computational design of a thermostable de novo biocatalyst for whole cell biotransformations. *bioRxiv* 617055 (2024).

76. Anishchenko, I. *et al.* Modeling protein-small molecule conformational ensembles with ChemNet. *bioRxiv* 2024.09.25.614868 (2024).

77. Bjelic, S. *et al.* Exploration of Alternate Catalytic Mechanisms and Optimization Strategies for Retroaldolase Design. *J. Mol. Biol.* **426**, 256–271 (2014).
